# The whole set of the constitutive promoters recognized by four minor sigma subunits of *Escherichia coli* RNA polymerase

**DOI:** 10.1371/journal.pone.0179181

**Published:** 2017-06-30

**Authors:** Tomohiro Shimada, Kan Tanaka, Akira Ishihama

**Affiliations:** 1 Research Center for Micro-Nano Technology, Hosei University, Koganei, Tokyo, Japan; 2 Laboratory for Chemistry and Life Science, Institute of Innovative Research, Tokyo Institute of Technology, Nagatsuda, Yokohama, Japan; Indian Institute of Science, INDIA

## Abstract

The promoter selectivity of *Escherichia coli* RNA polymerase (RNAP) is determined by the sigma subunit. The model prokaryote *Escherichia coli* K-12 contains seven species of the sigma subunit, each recognizing a specific set of promoters. For identification of the “constitutive promoters” that are recognized by each RNAP holoenzyme alone in the absence of other supporting factors, we have performed the genomic SELEX screening *in vitro* for their binding sites along the *E*. *coli* K-12 W3110 genome using each of the reconstituted RNAP holoenzymes and a collection of genome DNA segments of *E*. *coli* K-12. The whole set of constitutive promoters for each RNAP holoenzyme was then estimated based on the location of RNAP-binding sites. The first successful screening of the constitutive promoters was achieved for RpoD (σ^70^), the principal sigma for transcription of growth-related genes. As an extension, we performed in this study the screening of constitutive promoters for four minor sigma subunits, stationary-phase specific RpoS (σ^38^), heat-shock specific RpoH (σ^32^), flagellar-chemotaxis specific RpoF (σ^28^) and extra-cytoplasmic stress-response RpoE (σ^24^). The total number of constitutive promoters were: 129~179 for RpoS; 101~142 for RpoH; 34~41 for RpoF; and 77~106 for RpoE. The list of constitutive promoters were compared with that of known promoters identified *in vivo* under various conditions and using varieties of *E*. *coli* strains, altogether allowing the estimation of “inducible promoters” in the presence of additional supporting factors.

## Introduction

The genome of *Escherichia coli* K-12, the most well-characterized model prokaryote, contains a total of more than 4,500 genes, which are transcribed by a single species of the RNA polymerase (RNAP). The intracellular concentration of RNAP is, however, approximately 2,000 molecules per genome, which is less than the total number of genes or operons [1–3]. The pattern of genome expression is therefore determined by the selective distribution of a limited number of RNAP within the genome [4,5]. For adaptation to stressful environments, the pattern of genome transcription is, however, altered by modulating the promoter selectivity of RNAP through two-step interaction with two groups of the regulatory factor, *i*.*e*., 7 species of the sigma factor with promoter recognition activity at the first step [5,6] and then approximately 300 species of the transcription factor (TF) including both protein and nucleotide factors at the second step [4,5,7,8]. For understanding the genome regulation at molecular level, therefore, three kinds of the basic knowledge are absolutely needed for both all the sigma and TF factors [8,9]: (1) the whole set of regulatory target promoters, genes or operons under the control of each regulatory factor; (2) the binding affinity of the test regulatory protein to target DNA; and (3) the intracellular concentrations of the functional forms of each regulatory protein [note that the activity of TF is often controlled by effector ligands or protein modification such as phosphorylation]. Once we get these three lines of knowledge, we will be able to predict the pattern of genome transcription.

After the complete genome sequencing of *E*. *coli* K-12, its transcription pattern or transcriptome *in vivo* has been analyzed for various *E*. *coli* wild-type and mutant strains growing under various stress conditions, including niches within host animals, using modern technologies such as the microarray system [10,11]. The localization of RNAP and TFs on the genome was also analyzed by using ChIP-chip system [12–14]. More recently microarray was replaced by direct sequencing of RNAs [15–17] or mapping of transcription start sites [18,19]. These data are assembled in the databases such as RegulonDB [20,21] and EcoCyc [22,23]. The huge accumulation of background knowledge is absolutely needed for understanding the regulation mechanism of genome transcription as a whole in a single organism, and thus at this stage, *E*. *coli* is reassessed as the model organism. The binding sites of RNAP and TF identified *in vivo* using these modern techniques, however, do not represent the whole set of their binding sites because: i) their binding to regulatory sites is often interfered by other DNA-binding proteins, thereby masking their binding target sequences by antagonistic inhibitory proteins [8,9,24]; and ii) in the case of activator-dependent transcription, their binding to targets depends on the simultaneous presence of supporting factors [8,9,25]. Under the *in vivo* situations, therefore, it is in principle impossible to obtain the whole set of binding sites for both RNAP and TFs. In addition, the transcription-related data listed in the databases include different levels of accuracy. For instance, a number of TF-binding sites are estimated in *silico* relying on the consensus sequences that often include the inaccurate prediction. Another serious problem is originated from the use of various *E*. *coli* strains with different genetic background and of different culture conditions used in each experiment (for details see Discussion).

In order to avoid the problems associated with these *in vivo* experiments, we then decided to employ the *in vitro* approaches. For identification of the binding sites of RNAP and TFs, we developed the Genomic SELEX system [26] and successfully employed for search of regulatory targets for a number of TFs [8,9]. We also employed the Genomic SELEX for mapping of promoters. As described in the previous report [27], we identified a total of 2,071 sites on the *E*. *coli* K-12 genome of binding of RNAP holoenzyme containing RpoD (σ^70^), the major sigma for transcription of most of the growth-related genes, and mapped the location of “constitutive promoters” that are recognized by RpoD holoenzyme alone in the absence of other DNA-binding proteins [Note that the “constitutive promoter” is defined as the promoter that is recognized by RNAP alone in the absence of supporting factors while the promoters that are detected only *in vivo* are defined as the “inducible promoters”, supposedly under the support of accessory regulatory factors].

Besides this major house-keeping RpoD sigma (σ^70^), *E*. *coli* K-12 contains six alternative minor sigma factors, *i*.*e*., nitrogen-regulated gene-specific RpoN (σ^54^), stationary-phase nutrient-starvation specific RpoS (σ^38^), heat-shock response-specific RpoH (σ^32^), flagellar-chemotaxis specific RpoF (σ^28^), extra-cytoplasmic stress-response RpoE (σ^24^), and iron-starvation specific FecI (σ^28^) [4–6]. In this study, we identified the list of constitutive promoters for four minor sigma factors, RpoS, RpoH, RpoF and RpoE. Since RpoN sigma requires an additional TF such as NtrC for promoter binding, the set of promoters recognized by RpoN sigma differs depending on the species of collaborative TF. The list of promoters recognized by RpoN will be described elsewhere. On the other hand, FecI sigma is rather a unique sigma that recognizes only a specific target of the gene for *fecA* encoding transport of ferric citrate [28]. Thus, these two sigma factors, RpoN and FecI, are not included in this report. The list of constitutive promoters herein described provides the fundamental catalogs for the promoters recognized by the four minor sigma factors alone. The data described in this report will be deposited into TEC (Transcription Profile of *Escherchia coli*) database (https://shigen.nig.ac.jp/ecoli/tec/) [9]. The data of each minor sigma will be shown by ordering the sigma name (RpoS, RpoH, RpoF or RpoE) [https://shigen.nig.ac.jp/ecoli/tec/tfmap]. For details follow the instruction in TEC [9].

## Results

### Genomic SELEX screening for the constitutive promoters

The constitutive promoters are transcribed *in vitro* by the RNA polymerase holoenzyme alone in the absence of supporting factors. In order to identify the whole set of constitutive promoters on the entire genome of *E*. *coli* K-12 W3110, we performed a mass-screening *in vitro* of the whole set of sequences that are recognized by the reconstituted holoenzymes, each containing only one specific minor sigma factor. The sigma-free core enzyme was prepared by passing the purified RNA polymerase three times through phosphocellulose column chromatography in the presence of 5% glycerol, the stabilizer of holoenzyme complexes in the storage buffer [29]. The level of remaining sigma subunits was less than 0.1%, if any, as detected by both protein staining and immuno-staining against each of all seven species of sigma factors (RpoD, RpoN, RpoS, RpoH, RpoF, RpoE and FecI). The stoichiometry between core enzyme subunits was also checked by immune-staining with antibodies against the core subunits, RpoA, RpoB, RpoC and RpoZ. The holoenzymes fully saturated with each sigma subunit were reconstituted by mixing this sigma-free core enzyme and 4-fold molar excess of purified sigma factors, RpoS, RpoH, RpoF and RpoE. Since these sigma subunits alone are unable to bind to promoter DNA, the presence of excess sigma does not interfere with the function of RNAP holoenzymes. For the identification of DNA sequences that are recognized by each holoenzyme, we employed the Genomic SELEX screening system [26], in which a library of *E*. *coli* genome DNA fragments of 200–300 bp in length was used instead of synthetic oligonucleotides with all possible sequences used in the original SELEX method [30–32].

The multi-copy plasmid library of 200–300 bp-long random DNA fragments was constructed from the *E*. *coli* K-12 W3110 genome [26]. The library used in this study contained 6.5-fold molar excess of the entire genome, and thus a single and the same sequence might be included in 6 different overlapping segments on average, thereby increasing the resolution of mapping of SELEX fragments. In each experiment of Genomic SELEX screening, the mixture of genome DNA fragments, which was regenerated by PCR from the genome DNA library, was mixed with 2-fold molar excess of the reconstituted each RNAP holoenzyme, and subjected to Genomic SELEX screening. DNA-holoenzyme complexes formed were recovered using the anti-RpoC antibody, which gave the highest level of RNAP recovery among all the anti-core subunit antibodies. RNA polymerase-associated DNA was isolated from the antibody precipitates, amplified by PCR, and subjected to next cycles of SELEX. After repeated SELEX screening, the final products of holoenzyme-bound DNA fragments were subjected to mapping on the genome using a DNA tilling microarray (Oxford Gene Technology, Oxford, UK) [14]. The binding intensity was measured as the ratio of holoenzyme-bound DNA labeled by Cy3 against original library DNA labeled by Cy5 on an array and plotted along *E*. *coli* genome about each holoenzyme. On the DNA tilling array used, the 60 b-long probes are aligned along the *E*. *coli* genome at 105 bp-intervals, and therefore approximately 300 bp-long SELEX fragments should bind to two or more consecutive probes. This criterion was employed to avoid the background noise of non-specific binding of holoenzyme-bound DNA fragments to the tilling array [note that peaks showing hybridization to only a single probe was judged as a false-positive noise].

The binding sites were classified into two groups, one ‘within spacers’ and another ‘inside genes’. The binding sites on ‘within spacers’ were further classified into 3 types; type-A spacer located between bidirectional transcription units, type-B spacer located upstream of one transcription unit but downstream of another transcription unit, and type-C spacer located downstream of both transcription units. Based on the transcription direction of flanking genes, the total number of the constitutive promoters was predicted to range between the minimum [number of type-A spacer plus number of type-B spacer] and the maximum [number of type-A spacer x 2 plus number of type-B spacer]. The intragenic binding site was referred to type-D site. The height of binding intensity identified by SELEX-chip system is generally in good agreement with the number of clones identified by SELEX-clos (cloning-sequencing) system, indicating that these two parameters correlate with the binding affinity of test TF to DNA [4,5,8,9].

### The whole set of constitutive promoters for the stationary-phase sigma RpoS

In laboratory culture of *E*. *coli*, cell growth enters into the stationary phase mainly due to the limited availability of nutrients. Upon entry into the stationary phase, the pattern of genome expression is markedly altered by turning down the growth-related genes and instead up-regulation of the stress-response genes. In switching the transcription pattern, the stationary-phase specific minor sigma RrpoS is involved [33,34]. The *rpoS* gene is not essential for growth under non-stress conditions, but strains carrying mutations affecting *rpoS* activity are extremely sensitive to environmental stresses. As in the case of other sigma factors, RpoS interacts with RNAP core enzyme and modulates its promoter recognition specificity so as to recognize a specific but large set of genes.

**Fig 1:**
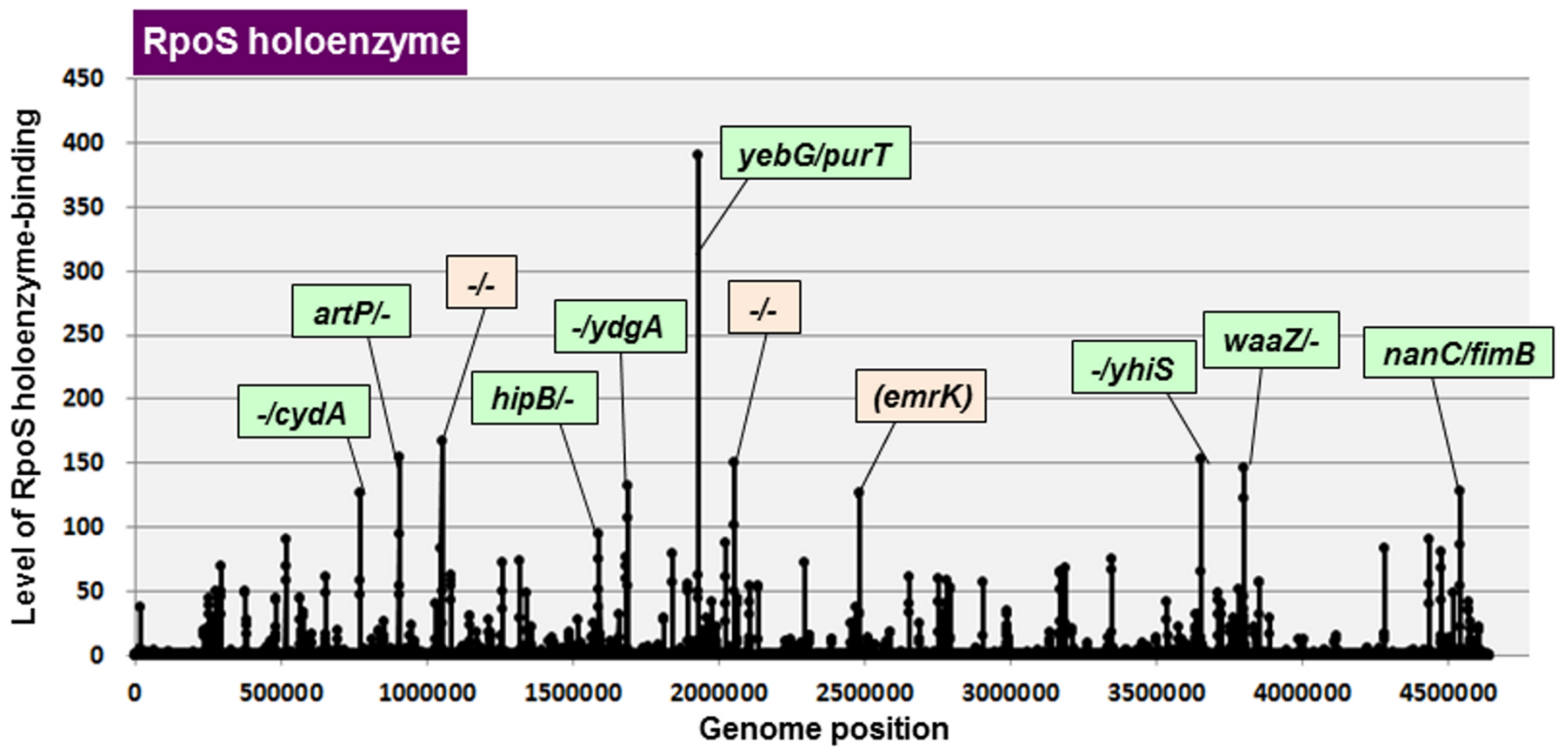
SELEX-chip search for RNAP RpoS holoenzyme-binding sequences on the *E*. *coli* K-12 genome. The y-axis represents the relative number of RpoS holoenzyme-bound DNA fragments whereas x-axis represents the position on the *E*. *coli* K-12 genome, in base pair. The adjacent gene on *E*. *coli* K-12 genome of peak position was indicated for high intensity peaks. The peaks located within spacer regions are shown in green color, while peaks located within open reading frames are shown in orange color. The list of RpoS holoenzyme-binding sites is described in Table 1.

As noted above, the set of genes identified *in vivo* include a number of genes under the indirect control of RpoS. On the other hand, some target promoters of RpoS are masked *in vivo* due to competitive interference by other DNA-binding proteins. In order to identify the constitutive promoters directly recognized by RpoS in the absence of other DNA-binding proteins, the Genomic SELEX screening *in vitro* was performed using the reconstituted RNAP RpoS holoenzyme. The sequences with binding affinity to the RpoS holoenzyme formed a number of peaks along the entire *E*. *coli* genome (Fig 1). Location of peaks was aligned along the map of *E*. *coli* K-12 genome (Table 1). By setting the cut-off level of 3.0 fold-higher intensity over the background of original library DNA, a total of 218 peaks were identified, of which 125 (67%) are located within intergenic spacers and 73 (33%) are inside of open reading frames (Table 2). Since the majority of hitherto identified promoters are located within spacers and generally upstream of open reading frames, detailed search for the constitutive promoters was focused on these 125 spacer peaks. These spacers can be classified into three types: 50 peaks are located within type-A spacer between bidirectional transcription units; 79 peaks are located within type-B spacers located upstream of one transcription unit but downstream of another transcription unit; and 16 peaks are located within type-C located spacers downstream of both transcription units. Based on the transcription direction of flanking genes, the total number of RpoS constitutive promoters was predicted to range between minimum 129 (50 type-A plus 79 type-B) and maximum 179 (50x2 type-A plus 79 type-B) (Table 2). Type-A spacers should contain two promoters for bidirectional transcription, at least one of which should be RpoS-dependent promoter. The RpoS holoenzyme-binding sites identified in a total of 50 type-A spacers should represent promoters for one or both of bidirectional transcription.

**Table 1:** RpoS holoenzyme-binding sites on the *E*. *coli* K-12 genome.

No	Type	Map	Gene Function	Left	D	RpoS	D	Right	Gene Function	Intensity
1	D	17732		***sokC***	>	***nhaA***	>	***nhaR***	transcriptional activator	5.3
2	A	41946	predicted transporter	***caiT***	<		>	***fixA***	electron transfer flavoprotein	3.6
3	B	63256	RNAP-associated helicase	***hepA***	<		<	***polB***		3.8
4	D	238050		***aspV***	>	***yafT***	<	***ykfM***		19.0
5	B	251970		***dinB***	>		>	***yafN***	YafO-YafN toxin-antitoxin system	45.1
6	B	289870	ornithine carbamoyltransferase 2	***argF***	<		<	***insB***		25.0
7	D	296234	CP4-6 prophage protein	***yagN***	<	***intF***	>	***ptwF***	Xaa tRNA	70.4
8	A	296336	CP4-6 prophage integrase	***intF***	<		>	***ptwF***	Xaa tRNA	45.5
9	A	328672	transcriptional repressor	***betI***	<		>	***betT***	choline transporter	3.8
10	B	379186	frmRAB operon regulator	***frmR***	<		<	***yaiO***		50.8
11	A	383868	conserved protein	***yaiS***	<		>	***tauA***	taurine transporter	24.1
12	D	451740	cytochrome o ubiquinol oxidase	***cyoA***	<	***ampG***	<	***yajG***		5.9
13	B	455746		***tig***	>		>	***clpP***	ATP-dependent serine protease	7.4
14	B	467530		***cof***	>		>	***ybaO***	DNA-binding transcriptional regulator	11.5
15	D	479868	modulator of gene expression with H-NS	***hha***	<	***tomB***	<	***acrB***		45.1
16	B	480454	predicted protein	***tomB***	<		<	***acrB***		22.6
17	A	515034	membrane anchored protease	***qmcA***	<		>	***ybbL***	ABC superfamily transporter	70.3
18	D	532860		***allA***	>	***allR***	>	***gcl***	glyoxylate carboligase	3.5
19	D	562966		***sfmH***	>	***sfmF***	<	***fimZ***		45.3
20	D	569366		***emrE***	>	***ybcK***	>	***ybcL***	DLP12 prophage kinase inhibitor	6.8
21	B	570042		***ybcK***	>		>	***ybcL***	DLP12 prophage kinase inhibitor	19.7
22	A	576032	IS5 transposase and trans-activator	***insH***	<		>	***essD***	DLP12 prophage lysis protein	34.0
23	B	582672		***nohB***	>		>	*appY	DLP12 prophage transcriptional activator	12.9
24	D	583166		***nohB***	>	***appY***	<	***ompT***		11.4
25	D	606570	predicted inner membrane protein	***ybdJ***	<	***ybdK***	>	***hokE***	toxic polypeptide	17.3
26	A	651466	citrate lyase synthetase	***citC***	<		>	***dpiB***	CitBA TCS sensory histidine kinase	8.3
27	D	654932		***dpiA***	>	***dcuC***	>	***pagP***	palmitoyl transferase for Lipid A	60.7
28	D	659134		***tatE***	>	***lipA***	<	***ybeF***		5.9
29	B	692668	nucleoside triphosphate hydrolase	***ybeZ***	<		<	***miaB***		19.9
30	C	720060		***ybfK***	>		<	***kdpE***		3.6
31	B	770146		***mngB***	>		>	***cydA***	cytochrome d terminal oxidase, subunit I	127.5
32	D	799356		***pgl***	>	***ybhD***	>	***ybhH***	conserved protein	4.8
33	A	812472	hypothetical protein	***ybhU***	<		>	***uvrB***	nucleotide excision repair nuclease	12.9
34	B	837732	conserved protein	*ybiI	<		<	***ybiX***		19.6
35	A	849568	threonine and homoserine efflux system	***rhtA***	<		>	***ompX***	outer membrane protein	5.4
36	A	852256	ncRNA	***rybA***	<		>	***mntR***	DNA-binding transcriptional regulator	26.0
37	A	877270	SAM-dependent methyltransferase	***yliG***	<		>	***bssR***	conserved protein	5.3
38	B	891170		***nfsA***	>		>	***rimK***	ribosomal protein S6 modification protein	4.1
39	B	903170	arginine transporter	*artP	<		<	***ybjP***		154.2
40	A	931668	thioredoxin reductase	***trxB***	<		>	***lrp***	DNA-binding transcriptional dual regulator	5.9
41	D	943972		***dmsB***	>	***dmsC***	<	***ycaC***		24.3
42	B	953940	formate transporter	***focA***	<		<	***ycaO***		3.4
43	B	959450		***aroA***	>		>	***ycaL***	peptidase with chaperone function	6.6
44	B	962934		***rpsA***	>		>	*ihfB	integration host factor (IHF)	3.6
45	D	1028042	hemimethhylated DNA-binding protein	***hspQ***	<	***yccW***	>	***yccX***	predicted acylphosphatase	40.5
46	C	1049830		***insB***	>		<	***cspH***		167.2
47	B	1084156		***efeB***	>		>	***phoH***	conserved protein with NTPase domain	62.7
48	B	1120372	predicted protein	***bssS***	<		<	***dinI***		6.6
49	B	1120772	DNA damage-inducible protein I	***dinI***	<		<	***pyrC***		3.7
50	D	1144848		***yceQ***	>	***rluC***	<	***yceF***		23.7
51	D	1147330		***rpmF***	>	***plsX***	>	***fabH***	3-oxoacyl-[acyl-carrier-protein] synthase III	31.2
52	A	1168238	DNA-binding transcriptional regulator	***ycfQ***	<		>	***bhsA***	predicted protein	17.6
53	D	1187848	conserved protein	***ycfD***	<	***phoQ***	<	***phoP***		5.5
54	D	1211030	5-methyl-C-specific restriction nuclease	***mcrA***	>	***elbA***	<	***ycgX***		4.7
55	D	1212068	predicted protein	***elbA***	<	***ycgX***	<	***ycgE***		27.8
56	A	1214962	predicted FAD-binding phosphodiesterase	***ycgF***	<		>	*ycgZ	predicted protein	14.2
57	B	1215830		***ariR***	>		>	***ymgC***	predicted protein	6.7
58	C	1218960		***ymgF***	>		<	***ymgD***		15.1
59	A	1250254	dihydroxyacetone kinase	*dhaK	<		>	*dhaR	DNA-binding transcriptional regulator	15.7
60	A	1257834	peptidyl-tRNA hydrolase	***pth***	<		>	***ychH***	predicted inner membrane protein	72.5
61	D	1318064	indole-3-glycerol-P synthetase	***trpC***	<	***trpD***	<	***trpE***		73.6
62	B	1341438	lipoprotein	*osmB	<		<	***yciT***		3.4
63	D	1342872	hypothetical protein	***yciZ***	<	***gmr***	<	***rnb***		48.2
64	A	1359040	gamma-Glu-putrescine synthase	*puuA	<		>	*puuD	gamma-Glu-GABA hydrolase	21.8
65	D	1393856		***mppA***	>	***ynaI***	>	***insH***	IS5 transposase and trans-activator	4.0
66	B	1432638	DNA-binding transcriptional regulator	***ynaE***	<		<	***uspF***		10.2
67	A	1438872	pyruvate-flavodoxin oxidoreductase	***ydbK***	<		>	***ydbJ***	predicted protein	5.1
68	D	1447730		***feaB***	>	***tynA***	<	***maoC***		5.0
69	C	1463278		***paaY***	>		<	***insD***		6.2
70	A	1489640	ncRNA	***rydC***	<		>	***ydcA***	predicted protein	14.9
71	B	1500460		***tehB***	>		>	***ydcL***	predicted lipoprotein	7.4
72	A	1515332	hypothetical protein	***yncL***	<		>	***ydcX***	predicted inner membrane protein	28.5
73	B	1518146		***yncB***	>		>	***mcbR***	DNA-binding transcriptional regulator	4.4
74	D	1528272		***yncH***	>	***ydcD***	>	***ydcC***	conserved protein	9.2
75	B	1543272	predicted protein	***yddJ***	<		<	***yddG***		3.5
76	D	1553260	dehydrogenase/acetaldehyde reductase	***adhP***	<	***maeA***	<	***sra***		4.9
77	D	1566238	predicted diguanylate cyclase	***yddV***	<	***yddW***	<	***gadC***		14.5
78	B	1570272	glutamate decarboxylase B	*gadB	<		<	***pqqL***		14.9
79	B	1580646	conserved protein	***ydeN***	<		<	***ydeO***		8.0
80	B	1590548	DNA-binding transcriptional regulator	***hipB***	<		<	***ydeU***		94.4
81	B	1596530	predicted lipoprotein	***ydeK***	<		<	***lsrK***		12.9
82	D	1613844		***yneJ***	>	***yneK***	>	***ydeA***	predicted arabinose transporter	4.3
83	B	1618030		***marA***	>		>	***marB***	predicted protein	3.9
84	B	1627238		***ydfH***	>		>	***ydfZ***	conserved protein	5.5
85	A	1630740	predicted mannonate dehydrogenase	***ydfI***	<		>	***ydfK***	Qin prophage transcriptional regulator	10.8
86	A	1655452	predicted protein	***ynfC***	<		>	***ynfD***	predicted protein	32.3
87	B	1682244		***rstB***	>		>	***tus***	inhibitor of replication at Ter	77.2
88	B	1687868		***manA***	>		>	***ydgA***	conserved protein	132.4
89	B	1745130		***ydhR***	>		>	***ydhS***	protein with FAD/NAD(P)-binding domain	3.8
90	A	1753168	predicted protein	***ydhZ***	<		>	*pykF	pyruvate kinase I	4.2
91	D	1769372	conserved protein	***ydiL***	>	***ydiM***	>	***ydiN***	predicted transporter	3.2
92	C	1793764	integration host factor (IHF)	***ihfA***	<	***pheT***	<	***pheS***		8.1
93	D	1811050		***ydjN***	>	***ydjO***	<	***cedA***		29.2
94	C	1841754		***gdhA***	>		<	***ynjI***		79.5
95	B	1894766		***nudL***	>		>	***sdaA***	L-serine deaminase I	55.4
96	B	1905768	predicted protein	***yobF***	<		<	***yebO***		11.2
97	D	1921260	ncRNA	***ryeA***	>	***ryeB***	<	***yebY***		11.5
98	B	1927030	protease II	***ptrB***	<		<	***yebE***		6.1
99	A	1928846	conserved protein regulated by LexA	***yebG***	<		>	***purT***	P-ribosylglycinamide formyltransferase 2	63.2
100	D	1928972	conserved protein regulated by LexA	***yebG***	<	***purT***	<	***eda***		390.6
101	A	1944202	RuvABC resolvasome	***ruvA***	<		>	***yebB***	predicted protein	11.3
102	B	1956162	TMAO reductase III (TorYZ)	***torY***	<		<	***cutC***		17.4
103	C	1966932	CheAB TCS chemotaxis regulator	***cheB***	<	***cheR***	<	***tap***		8.7
104	B	1994970	DNA-binding transcriptional activator	***sdiA***	<		<	***yecC***		21.7
105	A	2023030	predicted protein	*dsrB	<		>	***yodD***	predicted protein	88.4
106	B	2031954		***rseX***	>		>	*hchA	Hsp31 molecular chaperone	7.7
107	D	2049968		***asnT***	>	***yeeJ***	>	***shiA***	shikimate transporter	150.8
108	D	2057732	Asn tRNA	***asnW***	<	***yeeO***	>	***asnU***	Asn tRNA	3.2
109	B	2061434	L,D-transpeptidase linking Lpp to murein	***erfK***	<		<	***cobT***		44.9
110	D	2103732	predicted acyl transferase	***wbbJ***	<	***wbbI***	<	***rfc***		4.3
111	D	2104956	conserved protein	***wbbI***	<	***rfc***	<	***glf***		14.6
112	D	2106934	UDP-galactopyranose mutase	***glf***	<	***rfbX***	<	***rfbC***		54.2
113	B	2134130	protein-tyrosine phosphatase	***wzb***	<		<	***wza***		53.9
114	B	2226932	NAD(P)-binding oxidoreductase	***yohF***	<		<	***dusC***		3.3
115	A	2247636	DNA-binding transcriptional regulator	***yeiE***	<		>	***yeiH***	conserved inner membrane protein	12.7
116	D	2255672	predicted nucleoside transporter	***psuT***	<	***psuG***	<	***psuK***	pseudouridine kinase	7.7
117	B	2284170		***yejM***	>		>	***proL***	Pro tRNA	9.5
118	A	2311066	outer membrane porin protein C	***ompC***	<		>	***micF***	ncRNA	16.1
119	B	2311354		***micF***	>		>	***rcsD***	RcsBC TCS phosphotransfer protein	13.8
120	D	2389234		***yfbP***	>	***nuoN***	<	***nuoM***		13.0
121	B	2454170	predicted fimbrial-like adhesin protein	***yfcV***	<		<	***sixA***		25.4
122	D	2467360		***yfdH***	>	***yfdI***	<	***yfdK***		38.3
123	C	2468764		***yfdI***	>		<	***yfdK***		26.5
124	D	2480972	predicted multidrug efflux system	***emrY***	<	***emrK***	>	*evgA	EvgAS TCS response regulator	126.6
125	A	2510860	manganese/divalent cation transporter	***mntH***	<		>	***nupC***	nucleoside (except guanosine) transporter	13.5
126	D	2532356		***ptsH***	>	***ptsI***	>	*crr	glucose-specific PTS enzyme IIA	9.2
127	B	2574144	carboxysome structural protein	***eutS***	<		<	***maeB***		11.3
128	D	2587966		***narQ***	>	***acrD***	<	***ypfM***		18.4
129	B	2651536		***sseA***	>		>	***ryfA***	ncRNA	40.6
130	B	2689548	ncRNA	***glmY***	<		<	***purL***		24.9
131	B	2753630		***smpB***	>		>	***ssrA***	tmRNA	60.7
132	B	2763338	CP4-57 prophage; predicted protein	***yfjL***	<		<	***yfjM***		13.8
133	B	2765760		***yfjO***	>		>	***yfjP***	CP4-57 prophage GTP-binding protein	5.2
134	D	2771468		***yfjT***	>	***yfjW***	>	***yfjX***	CP4-57 prophage antirestriction protein	32.6
135	D	2772262		***yfjT***	>	***yfjW***	>	***yfjX***	CP4-57 prophage antirestriction protein	26.4
136	B	2773166		***yfjW***	>		>	***yfjX***	CP4-57 prophage antirestriction protein	41.0
137	D	2779640		***psaA***	>	***ypjA***	<	***ileY***		28.8
138	B	2783272	adhesin-like autotransporter	***ypjA***	<		<	***ileY***		58.9
139	A	2784466	Ile tRNA	***ileY***	<		>	*csiD	predicted protein	3.7
140	A	2786358	Ile tRNA	***ileY***	<		>	*csiD	predicted protein	31.8
141	A	2795168	predicted membrane protein	***yqaE***	<		>	***ygaV***	DNA-binding transcriptional regulator	53.2
142	B	2882250	predicted protein	***ygcL***	<		<	***ygcB***		6.3
143	A	2903434	conserved protein	***ygcF***	<		>	***ygcG***	predicted protein	57.0
144	B	2985568		***yqeG***	>		>	***yqeH***	protein with bipartite regulator domain	32.6
145	C	2987942		***yqeJ***	>		<	***yqeK***		9.1
146	D	2990738		***ygeG***	>	***ygeH***	>	***ygeI***	predicted protein	6.8
147	C	2992070		***ygeI***	>		<	***insD***		6.4
148	C	2992950		***ygeI***	>		<	***insD***		6.9
149	C	2993358		***ygeI***	>		<	***insD***		13.2
150	B	3134436	phosphate transporter	***pitB***	<		<	***gsp***		12.6
151	A	3145934	predicted protein	***yghW***	<		>	***yghZ***	aldo-keto reductase	3.2
152	B	3166762	predicted cyanide hydratase	***mqsR***	<		<	***ygiV***		65.1
153	C	3181642		***zupT***	>		<	***ribB***		10.0
154	B	3183246		***yqiC***	>		>	***ygiL***	predicted fimbrial-like adhesin protein	3.7
155	D	3189250		***yqiH***	>	***yqiI***	<	***glgS***		68.5
156	D	3210472		***rpsU***	>	***dnaG***	>	***rpoD***	RNA polymerase sigma 70	21.3
157	D	3259830	L-PSP (mRNA) endoribonuclease	***tdcF***	<	***tdcE***	<	***tdcD***		3.6
158	D	3266232		***tdcR***	>	***yhaB***	>	***yhaC***	predicted protein	9.6
159	C	3326238		***yhbY***	>		<	***greA***		3.6
160	B	3335948	ABC-type organic solvent transporter	***yrbC***	<		<	***yrbD***		14.0
161	D	3345766		***ptsN***	>	***yhbJ***	>	***npr***	N-regulated PTS system (Npr)	75.4
162	D	3348770	ncRNA	***ryhA***	>	***arcB***	<	***yhcC***		5.9
163	D	3395840	ribonuclease G	***rng***	<	***yhdE***	<	***mreD***		4.3
164	D	3453834	general secretory pathway component	***gspA***	<	***gspC***	>	***gspD***	general secretory pathway component	9.3
165	B	3467930	periplasmic endochitinase	***chiA***	<		<	***tufA***		5.0
166	B	3497370		***cysG***	>		>	***yhfL***	conserved secreted peptide	13.0
167	D	3533142		***pck***	>	***envZ***	<	***ompR***		41.3
168	D	3542562		***yhgA***	>	***bioH***	>	***gntX***	gluconate periplasmic binding protein	15.9
169	B	3576742	DNA-binding transcriptional repressor	***gntR***	<		<	***yhhW***		12.2
170	B	3582734		***yrhD***	>		>	***yrhB***	predicted protein	4.3
171	A	3584864	gamma-glutamyltranspeptidase	***ggt***	<		>	***yhhA***	conserved protein	12.9
172	D	3604756		***yhhN***	>	***zntA***	<	***sirA***		8.8
173	B	3621930		***yhhH***	>		>	***yhhI***	predicted transposase	3.3
174	A	3632156	predicted protein	***yhiJ***	<		>	*yhiM	conserved inner membrane protein	32.2
175	A	3632742	predicted protein	***yhiJ***	<		>	*yhiM	conserved inner membrane protein	13.3
176	A	3637868	universal stress protein B	*uspB	<		>	***uspA***	universal stress global response regulator	22.7
177	D	3647644		***arsR***	>	***arsB***	>	***arsC***	arsenate reductase	31.8
178	C	3648872		***arsC***	>		<	***insH***		153.6
179	B	3656130		***hdeD***	>		>	***gadE***	DNA-binding transcriptional activator	9.1
180	B	3708672	predicted metal dependent hydrolase	***eptB***	<		<	***yhjX***		48.9
181	B	3717944		***yiaG***	>		>	***cspA***	major cold shock protein	6.1
182	B	3720058		***insK***	>		>	***sokA***	ncRNA	31.7
183	A	3749938	predicted protein	***yiaT***	<		>	***yiaU***	DNA-binding transcriptional regulator	22.4
184	B	3794944		***rfaC***	>		>	***rfaL***	O-antigen ligase	3.8
185	D	3796942		***rfaL***	>	***waaU***	<	***rfaZ***		46.2
186	D	3798472	lipopolysaccharide core synthesis protein	***rfaZ***	<	***rfaY***	<	***rfaJ***		145.8
187	D	3800958	UDP-D-glucose:LPS glucosyltransferase	***rfaJ***	<	***rfaI***	<	***rfaB***		15.8
188	D	3802448	UDP-galactose:LPS galactosyltransferase	***rfaB***	<	***rfaS***	<	***rfaP***		21.5
189	B	3834856	Sec tRNA	***selC***	>		>	***setC***	predicted sugar efflux system	22.6
190	A	3851272	ncRNA	***istR***	<		>	***tisB***	lexA-regulated toxic peptide	57.3
191	B	3886640		***tnaC***	>		>	***tnaA***	tryptophanase/L-cysteine desulfhydrase	29.5
192	D	4001054	conserved inner membrane protein	***yigF***	<	***yigG***	<	***rarD***		5.5
193	D	4002164	predicted inner membrane protein	***yigG***	<	***rarD***	<	***yigI***		13.0
194	B	4076572		***yiiD***	>		>	***yiiE***	predicted transcriptional regulator	4.5
195	D	4110740	conserved protein	***yiiQ***	<	***yiiR***	>	***yiiS***	conserved protein	10.8
196	A	4116232	glycerol facilitator	***glpF***	<		>	***yiiU***	conserved protein	15.6
197	B	4120330	HslUV protease	***hslV***	<		<	***ftsN***		3.6
198	D	4220334		***aceK***	>	***arpA***	<	***iclR***		5.4
199	B	4281230	conserved protein	***yjcF***	<		<	***actP***		83.6
200	A	4380530	fumarate reductase	***frdA***	<		>	***poxA***	predicted lysyl-tRNA synthetase	4.7
201	A	4417636	L-ascorbate 6-phosphate lactonase	***ulaG***	<		>	***ulaA***	L-ascorbate-specific PTS enzyme IIC	4.9
202	A	4432132	NAD(P)H:quinone oxidoreductase	***ytfG***	<		>	***ytfH***	predicted transcriptional regulator	90.2
203	A	4434562	2':3'-cyclic-nucleotide 2'-phosphodiesterase	***cpdB***	<		>	***cysQ***	PAPS 3'(2'),5'-bisphosphate nucleotidase	4.3
204	B	4472740		***yjgJ***	>		>	***yjgK***	conserved protein	15.2
205	C	4475268		***yjgL***	>		<	***argI***		80.9
206	A	4477770	predicted acetyltransferase	***yjgM***	<		>	***yjgN***	conserved inner membrane protein	4.1
207	A	4492648	L-idonate 5-dehydrogenase, NAD-binding	***idnD***	<		>	***idnK***	D-gluconate kinase, thermosensitive	6.0
208	B	4504448		***yjhC***	>		>	***ythA***	expressed protein	8.4
209	C	4505142		***ythA***	>		<	***insI***		5.3
210	A	4538166	N-acetylnuraminic acid OM channel protein	***nanC***	<		>	***fimB***	Tyr recombinase/*fimA* inversion regulator	5.5
211	A	4538758	N-acetylnuraminic acid OM channel protein	***nanC***	<		>	***fimB***	Tyr recombinase/*fimA* inversion regulator	127.7
212	D	4543668		***fimC***	>	***fimD***	>	***fimF***	minor component of type 1 fimbriae	5.0
213	C	4570642		***yjiS***	>		<	***mcrC***		41.4
214	D	4575670		***yjiS***	>	***mcrC***	<	***mcrB***		12.1
215	D	4576468	5-methylcytosine-specific restriction enzyme	***mcrC***	<	***mcrB***	<	***symE***		26.3
216	D	4588370	conserved protein	***yjiX***	<	*yjiY	>	***tsr***	methyl-accepting chemotaxis protein I	10.5
217	A	4601330	predicted inner membrane protein	***yjjP***	<		>	***yjjQ***	DNA-binding transcriptional regulator	22.9
218	B	4609336		***prfC***	>		>	***osmY***	periplasmic protein	3.7

Genomic SELEX was performed for search of the binding sites of RNAP RpoS holoenzyme. By setting the cut-off level of 3.0, a total of 218 binding sites were identified (see Fig 1 for SELEX pattern), which are aligned along the map of *E*. *coli* K12 genome. A total of 125 sites are located within intergenic spacers: 50 within type-A spacers (shown under orange background); and 79 within type-B spacers (shown under green background). The constitutive promoters of RpoS were predicted based on the adjacent genes [note that only the genes next to the RpoS holoenzyme-binding sites are shown] and the gene orientation (shown by arrows in the column of transcription direction). A total of 73 RpoS holoenzyme-binding sites are located inside open reading frames as indicated by the gene symbols shown in RpoS column.

*The genes listed in RegulonDB as the regulated targets of RpoS.

**Table 2:** Distribution of the binding sites of each RNAP holoenzyme.

Sigma	Total no. holoenzyme binding sites	Within Spacers			Inside Genes	Constitutive promoters		
		Type-A	Type-B	Type-C		Type-A spacer	Type-B spacer	Total
**RpoD**	**1320**	**177**	**317**	**49**	**777 (60%)**	**177~354**	**317**	**494~671**
		**543 (40%)**						
**RpoS**	**218**	**50**	**79**	**16**	**73 (33%)**	**50~100**	**79**	**129~179**
		**125 (67%)**						
**RpoH**	**133**	**41**	**60**	**6**	**26 (20%)**	**41~82**	**60**	**101~142**
		**107 (80%)**						
**RpoF**	**105**	**7**	**27**	**3**	**68 (65%)**	**7~14**	**27**	**34~41**
		**37 (35%)**						
**RpoE**	**126**	**29**	**48**	**7**	**42 (33%)**	**29~58**	**48**	**77~106**
		**84 (67%)**						

RNAP holoenzyme was reconstituted from the sigma-free core enzyme and 4-fold molar excess of each sigma subunit. The binding site of each holoenzyme on the genome of *E*. *coli* K-12 W3110 was determined *in vitro* using the improved Genomic SELEX screening system. Details of the experimental procedures are described previously [26]. The number of constitutive promoters were estimated based on the location of holoenzyme-binding sites. The number of constitutive promoters recognized by RpoD holooenzyme were described in the previous report [27].

Up to the present, two general approaches have been employed to define the RpoS regulon: the proteome analysis using two-dimensional gels of whole cell lysates [35]; and the transcriptome analysis using ChIP-chip or ChIP-Seq systems [37–39]. These studies altogether indicated that RpoS regulates, directly or indirectly, 10% (approximately 500 genes) of the *E*. *coli* genes, of which only about 140 genes were predicted to be under the direct control *in vivo* of RpoS [38]. The total number of RpoS promoters (or the transcription initiation sites) listed in the current RegulonDB database is as many as 164 [note that all these promoters were detected *in vivo*]. Of which 21 were identified by setting the cut-off level at 3.0 (Table 1, marked by asterisk), indicating that only these promoters represent the constitutive promoters and the majority of other known RpoS promoters represent the inducible promoters that are expressed only under the support of regulatory factors. Genomic SELEX analysis identified minimum 129 and maximum 179 RpoS constitutive promoters including 21 known RpoS-dependent promoters (Table 2). The highest peak (390-fold higher than the background of original library alone) was located at the 5’-proximal region of the *purT* gene (Fig 1), which encodes a bifunctional enzyme with both phosphoribosylglycinamide formyltransferase using formate (the third-step reaction of purine nucleotide synthesis) and acetate kinase for the synthesis of acetylphosphate (AcP). AcP might be utilized as the general phosphate donor for phosphorylation of most of the stress-response TCS (two-component system) response regulators under stressful conditions. A high-level peak (154-fold higher than the background of DNA library) was detected upstream of the *artPIQM* operon encoding L-arginine ABC transporter. This promoter was also identified *in vivo* to be RpoS dependent [36] (Table 1). High-level binding of the RpoS holoenzyme was also identified upstream of the *cydAB* operon encoding cytochrome oxidase for anaerobic respiration, and the *hipBA* operon encoding anti-toxin-toxin pair for control the persistence (Fig 1 and Table 1). RpoS-dependent constitutive promoter(s) also exists upstream of the *nanCMS* operon (N-acetylneuraminic acid transport and utilization) and/or the *fimB* gene (regulator for *fimA* encoding fimbrin, the major type-1 pili) (Fig 1 and Table 1). Noteworthy is that most of the RpoS-dependent promoters listed in the current databases might be those under the indirect control of RpoS [8,9,27]. Otherwise a set of RpoS-dependent promoters, designated as the inducible promoters, might be activated in the presence of additional supporting factors.

Using the newly constructed collection of *E*. *coli* promoters expressing two-fluorescent reporters, one attached to the test promoter and another to the reference promoter, we performed a systematic quantitative search *in vivo* for *E*. *coli* promoters that are activated in the stationary phase [39]. The activity of RpoS-dependent promoters was measured at various growth phases under various growth conditions. The results indicated that the constitutive promoters exhibited low but steady-state activity while the inducible promoters generally showed high activity during the transition from exponential growth to stationary phase.

The RpoS regulon is involved in not only cell survival in the stationary phase, but also in cross protection against various stresses, including nutrient starvation, osmotic stress, acid shock, cold shock, heat shock, and oxidative DNA damage [33,34]. Beyond entry into stationary phase, *E*. *coli* forms aggregates or biofilms that are morphologically and physiologically distinct from cells of planktonic growth. This requires coordinated production of an extracellular matrix of polysaccharide polymers and protein fibers that facilitate cell aggregation and adhesion to solid surface. The genes involved in biofilm formation and transformation into persister cells were included in the list of RpoS constitutive promoters [40,41].

### The whole sets of constitutive promoters for heat-shock response sigma RpoH

When *E*. *coli* cells are exposed to higher temperature, a set of heat-shock proteins (HSPs) is markedly and transiently induced. Heat shock-induced proteins (HSPs) play major roles in controlling the structure and function of various proteins, including protein folding, assembly, transport, repair and degradation during normal growth as well as under stress conditions [42,43]. The heat-shock response is a cellular protective system for maintenance of protein homeostasis. The set of HSPs include the GroEL (HSP60) and DnaK (HSP70) chaperones and the Lon and the Clp proteases. RpoH is specifically required for expression of the genes encoding a set of HSPs as identified by proteome [44,45] and also by transcriptome analyses [46]. Genome-wide transcription profiling of the regulatory targets of RpoH was identified under the moderate induction of a plasmid-borne *rpoH* gene under defined, steady state growth conditions [47]. A total of 126 genes were influenced in the absence or in the over-expression of RpoH, which are organized in 85 operons. The set of genes identified *in vivo* by changing the level of RpoH include a large number of indirect targets, which are affected in response to the changes in the level of direct target [8,9,27]. The total number of RpoH promoters (or the transcription initiation sites) listed in the current RegulonDB database is as many as 322, but the majority of RpoH targets are predicted by the computational analysis using the consensus sequence that was predicted based on a few experimentally identified RpoH promoters.

**Fig 2:**
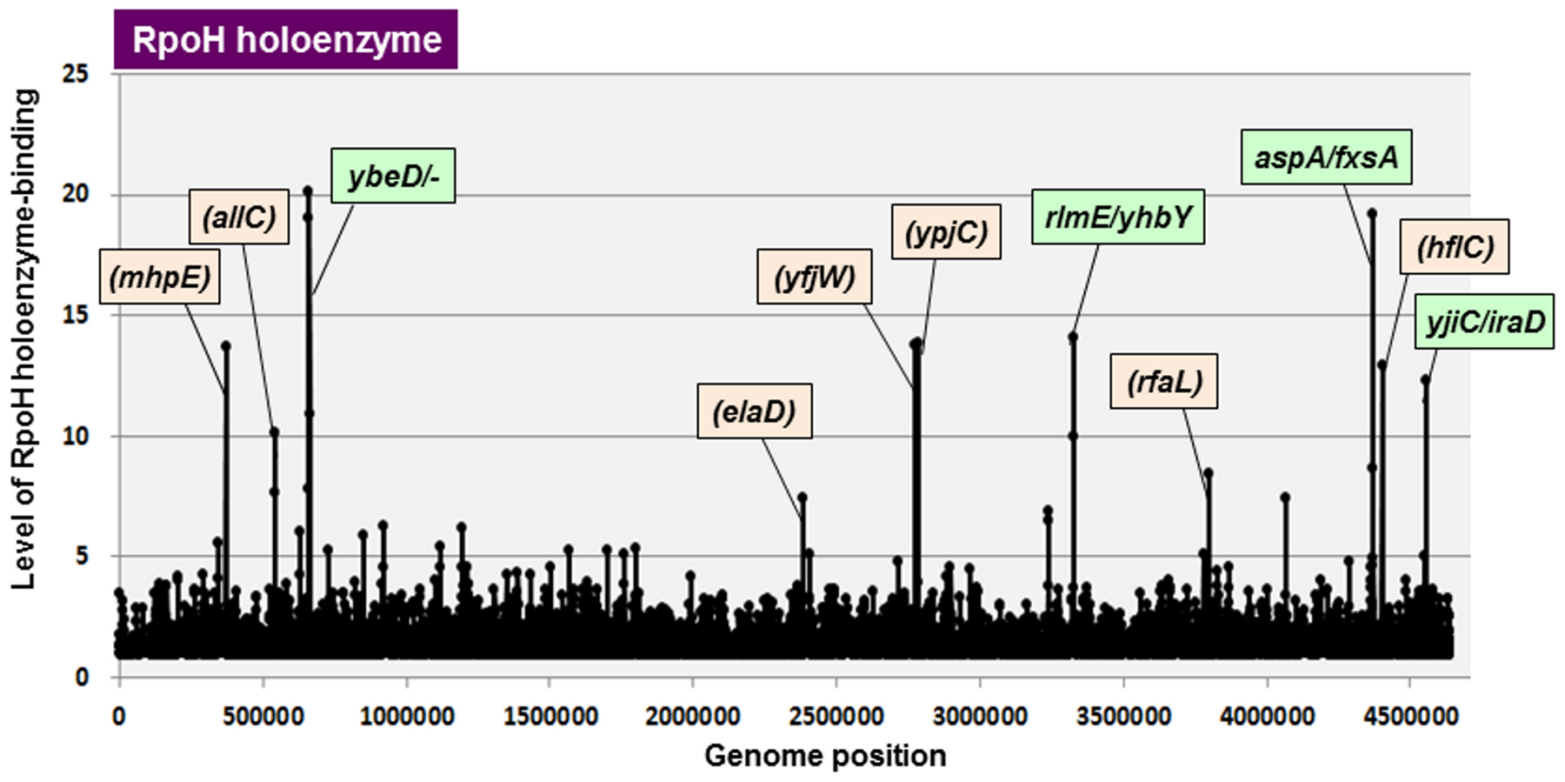
SELEX-chip search for RNAP RpoH holoenzyme-binding sequences on the *E*. *coli* K-12 genome. The y-axis represents the relative number of RpoH holoenzyme-bound DNA fragments whereas x-axis represents the position on the *E*.*coli* K-12 genome, in base pair. The adjacent gene on *E*. *coli* K-12 genome of peak position was indicated for high intensity peaks. The peaks located within spacer regions are shown in green color, while peaks located within open reading frames are shown in orange color. The list of RpoH holoenzyme-binding sites is described in Table 3.

We isolated RpoH protein for the first time and confirmed its recognition *in vitro* of the known HSP gene promoters [48]. Since then no serious examination *in vitro* has been performed to identify the RpoH function and it regulatory targets. To get insights into the regulatory role of RpoH sigma, we then performed in this study the Genomic SELEX screening using the reconstituted RNAP RpoH holoenzyme. By setting the cut-off level of 3.0 fold higher than the background of original library DNA, a total of 133 RpoH holoenzyme-binding peaks were identified (Fig 2 and Table 3), of which 107 (80%) are located within intergenic spacers and 26 (20%) are inside of open reading frames (Table 2). Since the majority of hitherto identified promoters are located within spacers, detailed search for the constitutive promoters was focused on the total of 107 peaks within spacers. The spacers containing RpoH holoenzyme-binding sites were also classified into three types (Tables 2 and 3 for the whole list): 41 peaks are located within type-A spacer; 60 peaks are located within type-B spacers; and 6 peaks are located within type-C spacers. Based on the transcription direction of flanking genes, the total number of RpoH constitutive promoters was predicted to range between minimum 101 (41 type-A plus 60 type-B) and maximum 142 (41x2 type-A plus 60 type-B) (Table 2).

**Table 3:** RpoH holoenzyme-binding sites on the *E*. *coli* K-12 genome.

No	Type	Map	Gene Function	Left	D	RpoH	D	Right	Gene Function	Intensity
1	A	12044	predicted protein	***yaaI***	<		>	*dnaK	chaperone Hsp70	3.2
2	A	142736	carbonic anhydrase	*can	<		>	***yadG***	ABC superfamily transporter	3.9
3	B	155442	outer membrane usher protein	***htrE***	<		<	***ecpD***		3.0
4	B	164658		***hrpB***	>		>	***mrcB***	glycosyl transferase and transpeptidase	3.8
5	B	202068		***lpxD***	>		>	***fabZ***	hydroxymyristol acyl carrier dehydratase	4.0
6	B	229134		***aspU***	>		>	***dkgB***	2,5-diketo-D-gluconate reductase B	3.0
7	C	262270		***thrW***	>		<	***ykfI***		3.6
8	B	292170	CP4-6 prophage protein	***yagK***	<		<	***yagL***		4.3
9	B	331456		***betT***	>		>	***yahA***	DNA-binding transcriptional regulator	3.5
10	D	343660		***yahK***	>	***yahL***	>	***yahM***	predicted protein	5.6
11	B	379186	*frmRAB* operon regulator	***frmR***	<		<	***yaiO***		3.0
12	B	406536		***yaiA***	>		>	***aroM***	conserved protein	3.6
13	B	477848	conserved inner membrane protein	***ylaB***	<		<	***ylaC***		3.3
14	D	543470	conserved protein	***ylbA***	<	***allC***	<	***allD***		10.1
15	B	557960		***sfmA***	>		>	***sfmC***	pilin chaperone	3.5
16	D	559456		***sfmC***	>	***sfmD***	>	***sfmH***	fimbrial-like adhesin protein	3.1
17	B	581644		***nohB***	>		>	***appY***	DLP12 transcriptional activator	3.8
18	B	592452	phage N4 receptor IM protein	***nfrB***	<		<	***cusS***		3.2
19	B	629042		***ybdB***	>		>	***cstA***	carbon starvation protein	6.0
20	D	655854	anaerobic C4-dicarboxylate transport	***dcuC***	<	***pagP***	>	***cspE***	DNA-binding transcriptional repressor	3.3
21	B	661936	conserved protein	*ybeD	<		<	***dacA***		20.1
22	D	732870		***ybfA***	>	*rhsC	>	***ybfB***	predicted inner membrane protein	3.2
23	D	747240	predicted regulator	***abrB***	<	***ybgO***	<	***ybgP***		3.5
24	B	784068	zinc efflux system	***zitB***	<		<	***ybgS***		3.5
25	A	784656	conserved protein	***ybgS***	<		>	***aroG***	D-arabino-heptulosonate-7P synthase	3.0
26	B	837732	conserved protein	***ybiI***	<		<	***ybiX***		3.1
27	B	913136	anaerobic terminal reductases	***hcp***	<		<	***ybjE***		3.8
28	A	918368	conserved protein	***ybjX***	<		>	***macA***	macrolide transporter	6.3
29	B	959450		***aroA***	>		>	***ycaL***	peptidase with chaperone function	3.2
30	B	985134	aspartate aminotransferase	***aspC***	<		<	***ompF***		3.4
31	A	1050632	CspA-family stress protein	*cspH	<		>	***cspG***	DNA-binding transcriptional regulator	3.6
32	A	1102554	DNA-binding transcriptional activator	***csgD***	<		>	***csgB***	curlin nucleator protein	4.0
33	B	1120230	predicted protein	*bssS	<		<	***dinI***		5.4
34	C	1195868		***icd***	>		<	***ymfD***		6.2
35	D	1198640	e14 prophage inner membrane protein	***ymfE***	<	***lit***	<	***intE***		3.8
36	D	1200062	e14 prophage integrase	***intE***	<	***xisE***	>	***ymfI***	e14 prophage; predicted protein	3.2
37	B	1218154		***ycgG***	>		>	***ymgF***	predicted protein	3.4
38	C	1219948		***ymgF***	>		<	***ymgD***		3.9
39	A	1233950	sodium:proton antiporter	***nhaB***	<		>	***fadR***	DNA-binding transcriptional regulator	3.1
40	B	1255834	predicted adhesin	***ycgV***	<		<	***ychF***		3.2
41	B	1308330	voltage-gated potassium channel	***kch***	<		<	***yciI***		3.7
42	B	1349272	enoyl-[acyl-carrier-protein] reductase	***fabI***	<		<	***ycjD***		4.3
43	A	1389946	predicted hydrolase	*ycjY	<		>	*ycjZ	DNA-binding transcriptional regulator	4.4
44	B	1432738	Rac prophage transcriptional regulator	***ynaE***	<		<	***uspF***		4.2
45	B	1486246		***ydcF***	>		>	***aldA***	aldehyde dehydrogenase A	3.7
46	B	1565470	predicted diguanylate cyclase	***yddV***	<		<	***yddW***		5.2
47	B	1568560	glutamate:aminobutyric acid antiporter	***gadC***	<		<	***gadB***		2.3
48	B	1580550	conserved protein	***ydeN***	<		<	***ydeO***		3.3
49	B	1585730	fimbrial-like adhesin protein	***ydeQ***	<		<	***ydeR***		3.7
50	B	1613766		***yneJ***	>		>	*yneK	predicted protein	3.0
51	A	1630638	mannonate dehydrogenase	***ydfI***	<		>	***ydfK***	Qin prophage transcriptional regulator	4.0
52	B	1639072	Qin prophage S lysis protein	***essQ***	<		<	***cspB***		3.3
53	A	1639660	Qin prophage cold shock protein	***cspB***	<		>	***cspF***	Qin prophage cold shock protein	3.7
54	B	1646444		***dicA***	>		>	*ydfA	Qin prophage protein	3.2
55	B	1669358		***ynfM***	>		>	***asr***	acid-shock periplasmic protein	3.7
56	B	1751846	predicted oxidoreductase	***ydhV***	<		<	***ydhY***		3.1
57	B	1762570	Fe-S cluster assembly protein	***sufA***	<		<	***rydB***		5.1
58	A	1801256	threonyl-tRNA synthetase	***thrS***	<		>	***yniD***	predicted protein	5.3
59	A	1801758	threonyl-tRNA synthetase	***thrS***	<		>	***yniD***	predicted protein	3.4
60	B	1990832	PG phosphate synthase	***pgsA***	<		<	***uvrC***		4.2
61	D	2055332		***amn***	>	***yeeN***	<	***asnW***		3.0
62	A	2060070	DNA-binding transcriptionall regulator	***nac***	<		>	***asnV***	Asn tRNA	3.2
63	B	2096360	LPS O-antigen length regulator	*cld	<		<	***ugd***		3.2
64	D	2104956	conserved protein	***wbbI***	<	*rfc	<	***glf***		3.4
65	A	2261530	fructose-specific PTS enzyme IIA	***fruB***	<		>	***setB***	lactose/glucose efflux system	3.3
66	B	2276452	predicted protein	***yejG***	<		<	***bcr***		3.1
67	A	2342846	hypothetical protein	***ypaB***	<		>	***nrdA***	ribonucleoside diphosphate reductase	3.4
68	D	2355640		***glpC***	>	***yfaD***	>	***ypaA***	predicted protein	3.4
69	D	2363146		***nudI***	>	***ais***	>	***arnB***	uridine aminotransferase	3.8
70	B	2403570	NADH:ubiquinone oxidoreductase	***nuoA***	<		<	***lrhA***		5.1
71	A	2405036	DNA-binding transcriptional regulator	***lrhA***	<		>	***yfbQ***	predicted aminotransferase	3.2
72	D	2476546	DNA-binding transcriptional regulator	***dsdC***	<	***dsdX***	>	***dsdA***	D-serine ammonia-lyase	3.6
73	D	2489940	predicted transporter	***yfdV***	<	***oxc***	<	***frc***		3.6
74	D	2490262	predicted oxalyl-CoA decarboxylase	***oxc***	<	***frc***	<	***yfdX***		3.6
75	A	2492430	predicted protein	***yfdX***	<		>	***ypdI***	lipoprotein for colanic acid biosynthesis	3.0
76	A	2493362	predicted inner membrane protein	***yfdY***	<		>	***lpxP***	palmitoleoyl-ACP acyltransferase	3.6
77	D	2532356		***ptsH***	>	***ptsI***	>	***crr***	glucose-specific PTS enzyme IIA	3.2
78	A	2597838	dihydrodipicolinate synthase	***dapA***	<		>	***gcvR***	DNA-binding transcriptional repressor	3.1
79	B	2599140		***bcp***	>		>	***hyfA***	hydrogenase 4, 4Fe-4S subunit	3.1
80	D	2626032		***ppx***	>	***yfgF***	>	***yfgG***	predicted protein	3.6
81	A	2696642	conserved protein	***yfhB***	<		>	***yfhH***	DNA-binding transcriptional regulator	3.3
82	A	2714742	pyruvate formate lyase subunit	***yfiD***	<		>	***ung***	uracil-DNA-glycosylase	4.8
83	A	2739338	D-arabino-heptulosonate-7P synthase	***aroF***	<		>	***yfiL***	predicted protein	3.5
84	A	2823870	lytic murein transglycosylase B	***mltB***	<		>	***srlA***	glucitol/sorbitol-specific PTS IIC	3.1
85	A	2837534	DNA-binding transcriptional repressor	***ascG***	<		>	***ascF***	cellobiose/arbutin/salicin PTS IIB-IIC	3.5
86	D	2884946	predicted protein	***ygcL***	<	***ygcB***	<	***cysH***		3.4
87	A	2898372	deoxygluconate dehydrogenase	***ygcW***	<		>	***yqcE***	predicted transporter	4.6
88	A	2932264	L-fuculose-1-phosphate aldolase	***fucA***	<		>	***fucP***	L-fucose transporter	3.3
89	B	2967056	nucleotide hydrolase	***rppH***	<		<	***ygdT***		4.5
90	A	2976930	diaminopimelate decarboxylase	***lysA***	<		>	***lysR***	DNA-binding transcriptional regulator	3.4
91	B	2985164		***yqeG***	>		>	***yqeH***	protein with bipartite regulator domain	3.3
92	D	2985970		***yqeG***	>	***yqeH***	>	***yqeI***	transcriptional regulator	3.2
93	D	2991152		***ygeG***	>	***ygeH***	>	***ygeI***	predicted protein	3.7
94	C	2991858		***ygeI***	>		<	***insD***		3.5
95	B	3067930	mechanosensitive channel	***mscS***	<		<	***fbaA***		3.0
96	B	3166268	DNA-binding transcriptional regulator	***ygiT***	<		<	***mqsR***		3.0
97	B	3237654		***alx***	>		>	***sstT***	sodium:serine/threonine symporter	6.9
98	D	3266066		***tdcR***	>	***yhaB***	>	***yhaC***	predicted protein	3.0
99	A	3276944	DNA-binding transcriptional regulator	***agaR***	<		>	***kbaZ***	tagatose 6-phosphate aldolase 1	3.6
100	B	3319952	predicted hydrolase, inner membrane	***yhbX***	<		<	***leuU***		3.2
101	A	3325832	23S rRNA methyltransferase	***rrmJ***	<		>	***yhbY***	predicted RNA-binding protein	14.1
102	D	3360232		***gltF***	>	*yhcA	>	***yhcD***	predicted outer membrane protein	3.2
103	B	3375554	stringent starvation protein A	***sspA***	<		<	***rpsI***		3.7
104	B	3387142	p-hydroxybenzoic acid efflux system	*aaeA	<		<	***aaeX***		3.1
105	A	3559934	thiosulfate:cyanide sulfurtransferase	***glpE***	<		>	***glpD***	sn-glycerol-3-phosphate dehydrogenase	3.5
106	D	3629568	predicted HlyD family secretion protein	***yhiI***	<	***yhiJ***	>	***yhiM***	conserved inner membrane protein	3.6
107	C	3634072		***yhiM***	>		<	***yhiN***		3.8
108	B	3655654		***hdeD***	>		>	***gadE***	DNA-binding transcriptional activator	4.1
109	C	3661646		***mdtF***	>		<	***gadW***		3.7
110	B	3681552	C4-dicarboxylic acid citrate transporter	***dctA***	<		<	***yhjK***		3.0
111	A	3717248	conserved protein	***yiaF***	<		>	***yiaG***	transcriptional regulator	3.0
112	B	3717858		***yiaG***	>		>	***cspA***	major cold shock protein	3.0
113	B	3720058		***insK***	>		>	***sokA***	ncRNA	3.0
114	A	3826772	glutamate transporter	***gltS***	<		>	***yicE***	predicted transporter	4.4
115	B	3834954		***selC***	>		>	***setC***	predicted sugar efflux system	3.4
116	A	3865668	heat shock chaperone	*ibpA	<		>	***yidQ***	conserved outer membrane protein	4.6
117	A	3939432	predicted transcriptional regulator	***yieP***	<		>	*rrsC	16S ribosomal RNA of *rrnC* operon	3.6
118	A	4002730	conserved protein	***yigI***	<		>	*pldA	outer membrane phospholipase A	3.7
119	B	4068432	predicted aldose-1-epimerase	***yihR***	<		<	***yihS***		7.5
120	B	4360430	DNA-binding transcriptional activator	***cadC***	<		<	***pheU***		4.0
121	B	4364770	C4-dicarboxylate antiporter	***dcuA***	<		<	***aspA***		4.6
122	A	4366568	aspartate ammonia-lyase	***aspA***	<		>	*fxsA	inner membrane protein	19.2
123	D	4371850		***yjeI***	>	*yjeJ	<	***yjeK***		4.9
124	D	4486746		***yjgQ***	>	***yjgR***	<	***idnR***		4.1
125	B	4494638		***leuX***	>		>	***insC***	KpLE2 phage IS2 element repressor	3.1
126	B	4518430	KpLE2 transcriptional regulator	***yjhU***	<		<	***yjhF***		3.1
127	B	4530030	KpLE2 phage endoglucanase	***sgcX***	<		<	***yjhP***		3.3
128	A	4538050	N-acetylnuraminic acid channel protein	***nanC***	<		>	***fimB***	tyrosine recombinase, *fimA* regulator	3.5
129	A	4538964	N-acetylnuraminic acid channel protein	***nanC***	<		>	***fimB***	tyrosine recombinase, *fimA* regulator	3.4
130	B	4540968		***fimE***	>		>	***fimA***	major type 1 subunit fimbrin (pilin)	3.4
131	A	4549330	fructuronate transporter	***gntP***	<		>	***uxuA***	mannonate hydrolase	5.0
132	D	4576468	5-methylcytosine restriction enzyme	***mcrC***	<	***mcrB***	<	***symE***		3.5
133	A	4633370	DNA-binding transcriptional activator	***rob***	<		>	*creA	conserved protein	3.3

Genomic SELEX was performed for search of the binding sites of RNAP RpoH holoenzyme. By setting the cut-off level of 3.0, a total of 133 binding sites were identified (see Fig 2 for SELEX pattern), which are aligned along the map of *E*. *coli* K12 genome. A total of 107 sites are located within intergenic spacers: 41 wihin type-A spacers (shown under orange background); and 60 within type-B spacers (shown under green background). The constitutive promoters of RpoH were predicted based on the adjacent genes [note that only the genes next to the RpoH holoenzyme-binding sites are shown] and the gene orientation (shown by arrows in the column of transcription direction). A total of 26 RpoH holoenzyme-binding sites are located inside open reading frames as indicated by the gene symbols shown in RpoH column.

* The genes listed in RegulonDB as the regulated targets of RpoH.

Among a total of 322 RpoH promoters (or the transcription initiation sites) listed in RegulonDB database, 20 were identified setting the cut-off level at 3.0 (Table 3, marked by asterisk). The majority of RpoH promoters in the database were suggested to belong to the inducible promoters that are expressed only under the support of other positive regulatory factors. Otherwise these RpoH promoters might represent the inaccurate prediction as note above. Genomic SELEX analysis identified minimum 100 and maximum 140 RpoH constitutive promoters including 18 known RpoH dependent promoters (Table 2). The highest peak was 20-fold intensity that was detected on promoter region of the *ybeD* gene, which encodes a conserved protein of unknown function under regulation of RpoH (Fig 2) [49], followed by high-level peaks at the *aspA-fxsA* and the *rlmJ-yhbY* intergenic regions. The *fxsA* gene encodes an inner membrane protein, which is involved in sensitivity control to bacteriophage T7 [49]. The *rlmE* gene encodes 23S rRNA 2’-O-ribose U2552 metyltransferase, and has been proposed to carry RpoH-dependent promoter [50]. The regulatory target of RpoH sigma identified by Genomic SELEX expands to a set of genes related to varieties of stress-response genes beyond the HSP genes. In fact, the genes for response to environmental insults such as ethanol, alkaline pH, and hyperosmotic shock and the genes for proteolysis and cell division have been indicated under the control of RpoH. The set of RpoH-regulon genes thus identified *in vivo*, however, vary depending on the culture conditions.

### The whole sets of constitutive promoters for the flagella-chemotaxis sigma RpoF

The bacterial flagellum is a complex organelle consisting of three distinctive structural parts, the basal body, the hook and the filament [51]. The synthesis, assembly and function of the flagellar and chemotaxis system require the expression of more than 50 genes, which are divided into three temporally regulated transcriptional classes based on the hierarchy of expression order: class-I (early), class-II (middle), and class-III (late) [52,53]. The class-1 (early) consists of a single operon including two genes, *flhD* and *flhC*, each encoding transcription factor FlhD and FlhC, respectively, which together form a complex, FlhD_2_-FlhC_2_ or FlhD_4_-FlhC_2_, that activates transcription of a set of class-2 (middle) genes, including both the *rpoF* sigma gene (renamed *fliA*) and the *flgM* gene encoding the anti-RpoF factor [51,52]. RpoF is the sigma factor for flagellar chemotaxis, which recognizes the promoters of motility and flagellar synthesis genes. The regulatory target of RpoF in *Salmonella* was identified to include a set of genes that were classified into the class-3 operons of the flagella regulon [54,55]. More than 30 genes have been proposed to carry promoters that are under the control of RpoF sigma, including a set of the structural genes for flagella formation, and the chemotaxis genes encoding sensor of environmental signals affecting the motility control [54,56]. With use the combination of ChIP-chip, ChIP-seq and RNA-seq systems, a more comprehensive screening was recently performed for identification of the regulatory targets of RpoF sigma in *E*. *coli* [57]. A total of 52 RpoF-binding sites were identified *in vivo* on the genome of exponentially growing *E*. *coli* K-12 MG1655 cells in a rich LB medium, with a considerable level of over-lapping with the hitherto identified target genes of the RpoF regulon. The total number of RpoF promoters (or the transcription initiation sites) listed in the current RegulonDB database is as many as 144, which have been identified *in vivo* using ChIP-chip and ChIP-RNA Seq analyses. Most of the targets predicted by the *in vivo* data, however, represent those indirectly affected upon knock-out of the *rpoF* gene or over-production of RpoF.

**Fig 3:**
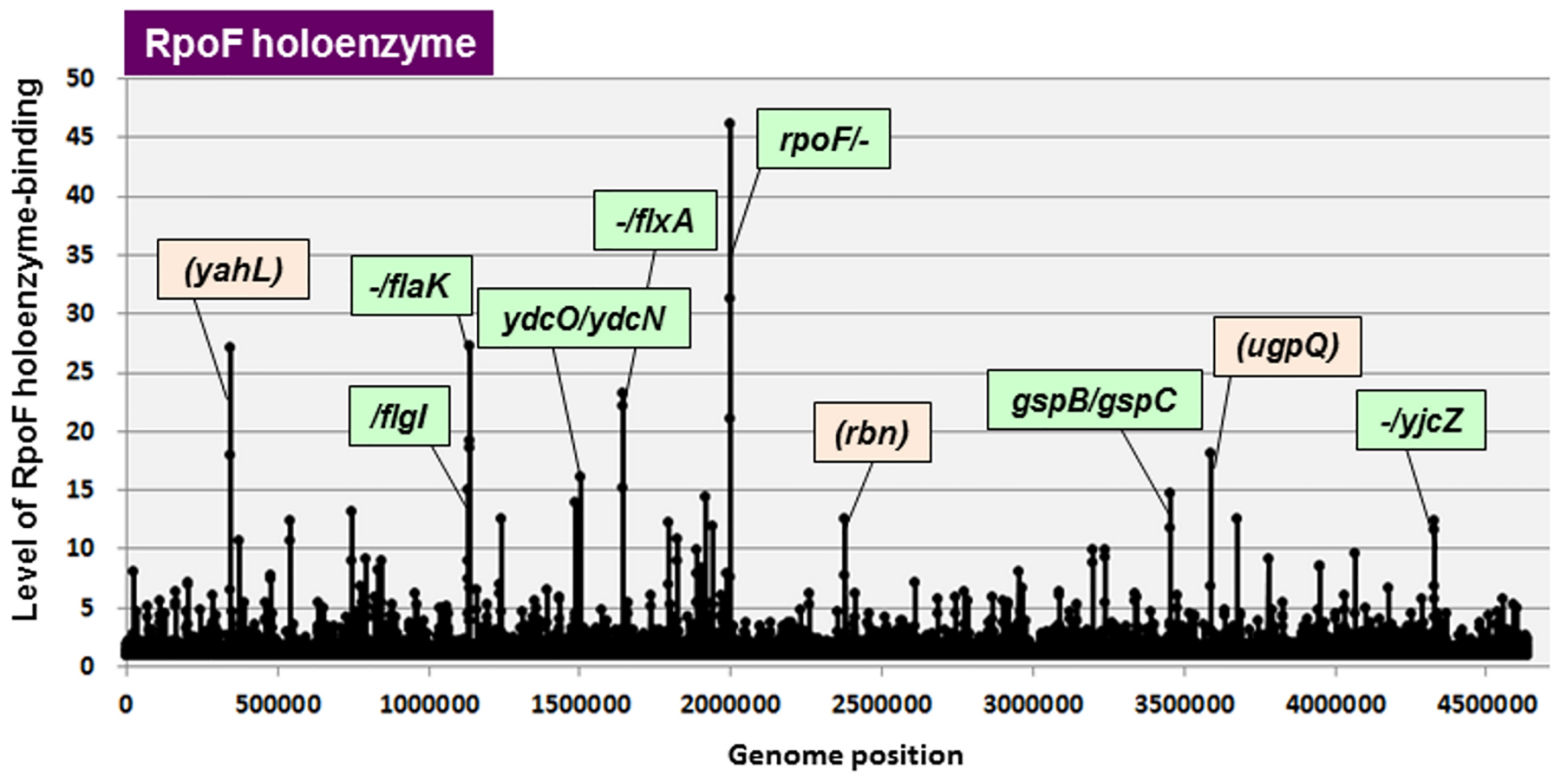
SELEX-chip search for RNAP RpoF holoenzyme-binding sequences on the *E*. *coli* K-12 genome. The y-axis represents the relative number of RpoF holoenzyme-bound DNA fragments whereas x-axis represents the position on the *E*. *coli* K-12 genome, in base pair. The adjacent gene on *E*. *coli* K-12 genome of peak position was indicated for high intensity peaks. The peaks located within spacer regions are shown in green color, while peaks located within open reading frames are shown in orange color. The list of RpoF holoenzyme-binding sites is described in Table 4.

We then performed the Genomic SELEX screening *in vitro* for search of the direct target promoters, genes and operons under the control of RpoF using the reconstituted RNAP RpoF holoenzyme. By setting the cut-off level of 4.0 fold higher than the background of original library DNA, a total of 105 RpoF holoenzyme-binding peaks were identified (Fig 3 and Table 4), of which 37 (35%) are located within intergenic spacers and 68 (65%) are inside of genes (Table 2). One unique feature of RpoF holoenzyme is its high-level (65%) binding to inside of open reading frames of a number of genes. A high-level (60%) of RNAP binding was also identified for RpoD holoenzyme [27]. The identification of the promoter-like sequences inside these genes awaits further analysis. The spacers containing RpoF holoenzyme-binding sites were also classified into three types (Tables 2 and 4 for the whole list): 7 peaks are located within type-A spacer; 27 peaks are located within type-B spacers; and 3 peaks are located within type-C spacers. Based on the transcription direction of flanking genes, the total number of RpoF constitutive promoters was predicted to range between minimum 34 (7 type-A plus 27 type-B) and maximum 41 (7x2 type-A plus 27 type-B) (Table 2). The total number of RpoF promoters (or the transcription initiation sites) listed in the current RegulonDB database is as many as 144. Of which 14 were identified setting cut-off level at 4.0 (Table 4, marked by asterisk), indicating that these promoters are constitutive promoters and the majority of known promoters represent the inducible promoters that are expressed only under the support of positive regulatory factors.

**Table 4:** RpoF holoenzyme-binding sites on the *E*. *coli* K-12 genome.

No	Type	Map	Gene Function	Left	D	RpoF	D	Right	Gene Function	Intensity
1	D	25672		***ileS***	>	***lspA***	>	***fkpB***	peptidyl-prolyl cis-trans isomerase	8.1
2	B	68342	L-arabinose isomerase	***araA***	<		<	***araB***		4.3
3	D	109556		***secM***	>	***secA***	>	***mutT***	NTP pyrophosphohydrolase	5.5
4	A	131366	predicted protein	***yacH***	<		>	***acnB***	aconitate hydratase	4.6
5	B	164658		***hrpB***	>		>	***mrcB***	glycosyl transferase and transpeptidase	6.4
6	D	198960		***rseP***	>	***bamA***	>	***skp***	periplasmic chaperone	4.7
7	B	202068		***lpxD***	>		>	***fabZ***	hydroxymyristol acyl carrier dehydratase	7.1
8	D	298660	conserved protein	***yagQ***	<	***yagR***	<	***yagS***		4.4
9	D	343660		***yahK***	>	***yahL***	>	***yahM***	predicted protein	27.1
10	D	353230		***prpD***	>	***prpE***	>	***codB***	cytosine transporter	4.7
11	B	463170		***ppiD***	>		>	***ybaV***	conserved protein	5.4
12	D	472938		***glnK***	>	***amtB***	<	***tesB***		4.0
13	D	477460		***ybaA***	>	***ylaB***	<	***ylaC***		7.8
14	D	482072	predicted protein	***tomB***	<	***acrB***	<	***acrA***		4.5
15	D	543470	conserved protein	***ylbA***	<	***allC***	<	***allD***		12.3
16	D	633930	predicted oxidoreductase	***ybdH***	<	***ybdL***	<	***ybdM***		5.4
17	D	652632	citrate lyase synthetase	***citC***	<	***dpiB***	>	***dpiA***	CitAB TCS response regulator	5.0
18	D	747240	predicted regulator	***abrB***	<	***ybgO***	<	***ybgP***		13.1
19	D	761630		***sucA***	>	***sucB***	>	***sucC***	succinyl-CoA synthetase	4.7
20	B	794336		***ybhT***	>		>	*modA	molybdate transporter subunit	9.2
21	B	822944	cardiolipin synthase 2	***ybhO***	<		<	***ybhP***		4.9
22	B	837732	conserved protein	***ybiI***	<		<	***ybiX***		5.7
23	D	844530		***rlmF***	>	***ybiO***	<	***glnQ***		9.0
24	B	874568		***yliE***	>		>	***yliF***	predicted diguanylate cyclase	4.3
25	D	878444		***bssR***	>	***yliI***	<	***yliJ***		5.3
26	D	893136		***ybjN***	>	***potF***	>	***potG***	ABC superfamily putrescine transporter	4.1
27	B	956734		***ycaP***	>		>	***serC***	3-phosphoserine aminotransferase	6.2
28	D	1039338		***appC***	>	***appB***	>	***yccB***	hypothetical protein	5.0
29	D	1048250	conserved protein	***gfcC***	<	***gfcB***	<	***gfcA***		4.0
30	B	1062060	modulator of CbpA co-chaperone	***cbpM***	<		<	***cbpA***		5.2
31	B	1129438	anti-sigma factor for FliA (sigma 28)	*flgM	<		<	***flgA***		15.0
32	B	1133868		***flgF***	>		>	***flgG***	flagellar component	6.7
33	B	1137530		***flgJ***	>		>	*flgK	flagellar hook-filament junction protein 1	27.2
34	D	1158658		***ptsG***	>	***fhuE***	>	***hinT***	purine nucleoside phosphoramidase	6.5
35	B	1193064	tRNA methyltransferase	***mnmA***	<		<	***nudJ***		5.3
36	D	1236068		***fadR***	>	***ycgB***	>	***dadA***	D-amino acid dehydrogenase	6.9
37	A	1243852	protein involved in flagellar function	*ycgR	<		>	***ymgE***	predicted inner membrane protein	4.6
38	B	1349272	enoyl-[acyl-carrier-protein] reductase	***fabI***	<		<	***ycjD***		5.6
39	D	1356550	predicted protein	***ymjA***	<	***puuP***	<	***puuA***		5.0
40	D	1392064		***ycjZ***	>	***mppA***	<	***ynaI***		6.5
41	A	1434934	outer membrane pore protein N	***ompN***	<		>	***micC***	ncRNA	5.9
42	B	1488236		***aldA***	>		>	***cybB***	cytochrome b561	13.9
43	D	1504230	predicted benzoate transporter	***ydcO***	<	***ydcN***	>	***ydcP***	predicted peptidase	16.1
44	B	1644248		***ydfV***	>		>	*flxA	Qin prophage; predicted protein	23.2
45	D	1661452		***ynfF***	>	***ynfG***	>	***ynfH***	oxidoreductase, membrane subunit	5.5
46	D	1734472		***sodB***	>	***ydhP***	<	***ynhF***		6.1
47	D	1794968	integration host factor (IHF)	***ihfA***	<	***pheT***	<	***pheS***		12.3
48	A	1801758	threonyl-tRNA synthetase	***thrS***	<		>	***yniD***	predicted protein	5.3
49	B	1815172	conserved protein	*chbG	<		<	***chbF***		4.2
50	D	1822736		***cho***	>	*ves	<	***spy***		10.8
51	D	1861032	methionine sulfoxide reductase B	***msrB***	<	***gapA***	>	***yeaD***	conserved protein	4.2
52	B	1887966	acyl-CoA synthetase	***fadD***	<		<	***yeaY***		9.9
53	A	1906860	predicted protein	***mgrB***	<		>	***yobH***	predicted protein	8.3
54	D	1914836	methionine-(R)-sulfoxide reductase	***yebR***	<	***yebS***	>	***yebT***	conserved protein	14.4
55	D	1928332	conserved protein	***yebE***	<	***yebF***	<	***yebG***		4.3
56	D	1938762	myristoyl-ACP-dependent acyltransferase	***lpxM***	<	***yebA***	<	***znuA***		11.9
57	D	1971862	purine-binding chemotaxis protein	***cheW***	<	***cheA***	<	***motB***		4.3
58	D	1988066	predicted protein	*yecH	<	***tyrP***	<	***yecA***		7.9
59	B	1999848	RNA polymerase sigma 28	*fliA	<		<	***fliC***		46.2
60	D	2233032		***yeiS***	>	***yeiT***	>	***yeiA***	Dihydropyrimidine dehydrogenase	4.8
61	D	2262454	fructose-specific PTS enzymes IIA	***fruB***	<	***setB***	<	***yeiW***		6.2
62	D	2379830	N-acyltransferase	***elaA***	<	***rbn***	>	***elaD***	predicted enzyme	12.5
63	B	2403570	NADH:ubiquinone oxidoreductase	***nuoA***	<		<	***lrhA***		3.5
64	D	2412454	conserved inner membrane protein	***yfbV***	<	***ackA***	>	***pta***	phosphate acetyltransferase	6.2
65	D	2456468	phosphohistidine phosphatase	***sixA***	<	***fadJ***	<	***fadI***		4.5
66	D	2609062		***hyfH***	>	***hyfI***	>	***hyfJ***	processing element hydrogenase 4	7.1
67	D	2684772	serine hydroxymethyltransferase	***glyA***	<	***hmp***	<	***glnB***		5.8
68	D	2745566	30S ribosomal subunit protein S16	***rpsP***	<	***ffh***	>	***ypjD***	predicted inner membrane protein	5.9
69	D	2868142	L-isoaspartate carboxymethyltransfeerase	***pcm***	<	***surE***	<	***truD***		5.8
70	D	2916840		***barA***	>	***gudD***	<	***gudX***		5.4
71	D	2952958	exonuclease V (RecBCD complex),	***recD***	<	***recB***	<	***ptrA***		8.0
72	D	2965066	diacylglyceryl transferase	***lgt***	<	***ptsP***	<	***rppH***		6.7
73	D	3087058		***metK***	>	***galP***	>	***yggI***	conserved protein	6.4
74	D	3124360	glycolate oxidase iron-sulfur subunit	***glcF***	<	***glcE***	<	***glcD***		4.0
75	C	3146946		***yghZ***	>		<	***yqhA***		5.2
76	B	3197666	deadenylyltransferase/adenylyltransferase	***glnE***	<		<	***ygiF***		8.8
77	B	3237654		***alx***	>		>	***sstT***	sodium:serine/threonine symporter	9.9
78	D	3338440	ABC superfamily toluene transporter	***yrbF***	<	***yrbG***	>	***kdsD***	D-arabinose 5-phosphate isomerase	6.2
79	D	3344772		***hpf***	>	***ptsN***	>	***yhbJ***	protein with NTP hydrolase domain	5.9
80	C	3388568		***aaeR***	>		<	***tldD***		4.7
81	D	3452166	type II secretion divergon	***gspB***	<	***gspA***	>	***gspC***	general secretory pathway component	11.8
82	D	3454168	general secretory pathway component	***gspA***	<	***gspC***	>	***gspD***	general secretory pathway component	14.7
83	D	3478072	predicted protein	***yheV***	<	***kefB***	<	***kefG***		6.1
84	D	3515848	predicted protein	***damX***	<	***aroB***	<	***aroK***		4.5
85	C	3528652		***hslO***	>		<	***yhgE***		4.0
86	D	3534454	TCS sensory histidine kinase	***envZ***	<	***ompR***	>	*greB	transcription elongation factor	4.3
87	D	3585866		***yhhA***	>	***ugpQ***	<	***ugpC***		18.1
88	D	3633746	predicted protein	***yhiJ***	<	*yhiM	<	***yhiN***		4.8
89	D	3689064	endo-1,4-D-glucanase	***bcsZ***	<	***bcsB***	<	***bcsA***		4.6
90	B	3779166		***lldD***	>		>	***yibK***	predicted rRNA methylase	9.2
91	D	3789732	threonine 3-dehydrogenase	***tdh***	<	***kbl***	<	***htrL***		4.9
92	A	3826672	glutamate transporter	***gltS***	<		>	***yicE***	predicted transporter	5.4
93	D	3829430		***yicE***	>	***yicH***	<	***yicI***		4.3
94	B	3906432	phosphate transporter	***pstB***	<		<	***pstA***		4.1
95	D	3945232		***trpT***	>	*hdfR	>	***yifE***	conserved protein	4.7
96	B	3951430		***ilvE***	>		>	***ilvD***	dihydroxyacid dehydratase	8.6
97	D	4003446	conserved protein	***yigI***	<	***pldA***	>	***recQ***	ATP-dependent DNA helicase	4.5
98	D	4032158		***yigZ***	>	***trkH***	>	***hemG***	protoporphyrin oxidase, flavoprotein	6.0
99	B	4068432	predicted aldose-1-epimerase	***yihR***	<		<	***yihS***		9.6
100	D	4178758		***rplJ***	>	***rplL***	>	***rpoB***	RNA polymerase, beta subunit	6.7
101	D	4286472	acetyl-CoA synthetase	***acs***	<	***nrfA***	>	***nrfB***	nitrite reductase	5.8
102	D	4327160	conserved protein	***yjdM***	<	*yjdA	>	***yjcZ***	conserved protein	12.4
103	D	4338862	biodegradative arginine decarboxylase	***adiA***	<	***melR***	>	***melA***	alpha-galactosidase, NAD(P)-binding	4.5
104	D	4535430	conserved protein	***yjhX***	<	***yjhS***	<	***nanM***		4.7
105	A	4589632	predicted inner membrane protein	***yjiY***	<		>	*tsr	methyl-accepting chemotaxis protein I	5.3

Genomic SELEX was performed for search of the binding sites of RNAP RpoF holoenzyme. By setting the cut-off level of 3.0, a total of 105 binding sites were identified (see Fig 3 for SELEX pattern), which are aligned along the map of E. coli K12 genome. A total of 37 sites are located within intergenic spacers: 7 wihin type-A spacers (shown under orange background); and 27 within type-B spacers (shown under green background). The constitutive promoters of RpoF were predicted based on the adjacent genes [note that only the genes next to the RpoF holoenzyme-binding sites are shown] and the gene orientation (shown by arrows in the column of transcription direction). A total of as many as 68 RpoF holoenzyme-binding sites are located inside open reading frames as indicated by the gene symbols shown in RpoF column.

*The genes listed in RegulonDB as the regulated targets of RpoF.

The highest peak was 46-fold intensity detected on promoter region of *rpoF* itself (Fig 3), indicating the autoregulation as already suggested [58]. A high-level peak was also identified upstream of the *flgK* gene, which encodes flagellar hook-filament junction protein that connects the filament to the hook, and its transcription has been shown *in vitro* under the direct control of RpoF [59]. The *flgM* gene encodes the anti-sigma factor for RpoF [55]. FlgM forms a complex with RpoF, thereby inactivating its sigma function but protects its degradation by the Lon protease for preservation [60].

### The whole set of constitutive promoters for extra-cytoplasmic stress response sigma RpoE

The bacterial cell envelope is a dynamic compartment, changing its structure and function in response to environmental conditions. Accordingly, the integrity of envelope is maintained through frequent modulation of its composition and components. The minor sigma factor RpoE plays a central role in this process, by controlling the selective expression of envelope components [61]. The regulatory targets have been estimated after proteome and transcriptome analyses *in vivo* [62–64]. The activity of RpoE is negatively regulated by a membrane-bound anti-sigma factor RseA, which sequesters RpoE under unstressed conditions. Within membrane, RseA is associated at its C-terminal domain with a periplasmic protein RseB, which senses misfolded proteins for release and activation of RpoE from RpoE-RseA complexes [62,65]. The total number of RpoE promoters (or the transcription initiation sites) listed in the current RegulonDB database is as many as 518, of which most are identified by computational analyses based on the consensus sequence of RpoE promoters without experimental analysis. After SELEX screening as noted below, most of these RpoE promoters must be inaccurate estimation due to the error in the consensus sequence.

**Fig 4:**
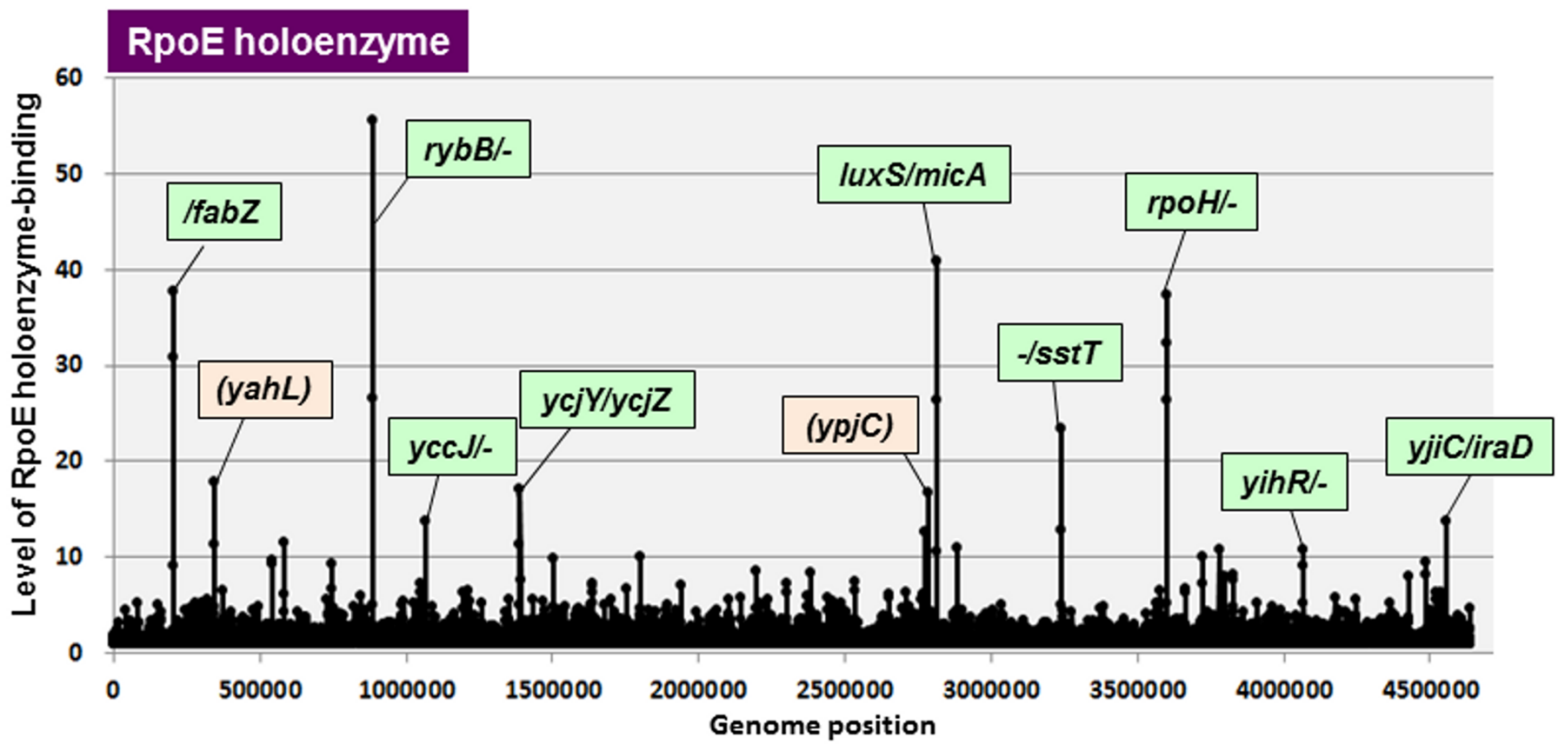
SELEX-chip search for RNAP RpoE holoenzyme-binding sequences on the *E*. *coli* K-12 genome. The y-axis represents the relative number of RpoE holoenzyme-bound DNA fragments whereas x-axis represents the position on the *E*.*coli* genome, in base pair. The adjacent gene on *E*. *coli* K-12 genome of peak position was indicated for high intensity peaks. The peaks located within spacer regions are shown in green color, while peaks located within open reading frames are shown in orange color. The list of RpoE holoenzyme-binding sites is described in Table 5.

The Genomic SELEX screening system was employed as a short-cut approach for identification of the RpoE regulon. Previously we purified RpoE and examined its promoter selectivity using an *in vitro* transcription system [66]. Using this purified RpoE, we performed SELEX screening. By setting the cut-off level of 4.0 fold against original library DNA, a total of 126 RpoE holoenzyme-binding peaks were identified (Fig 4 and Table 5), of which 84 (67%) are located within intergenic spacers and 42 (33%) are inside of open reading frames (Tables 2 and 5 for the whole set). Since the majority of hitherto identified promoters are located within spacers, detailed search for the constitutive promoters was focused on the total of 84 peaks within spacers. The spacers containing RpoE holoenzyme-binding sites were also classified into three types (Table 2): 29 peaks are located within type-A spacer; 48 peaks are located within type-B spacers; and 7 peaks are located within type-C spacers. Based on the transcription direction of flanking genes, the total number of RpoE constitutive promoters was predicted to range between minimum 77 (29 type-A plus 48 type-B) and maximum 106 (29x2 type-A plus 48 type-B) (Table 2). Within the set of constitutive promoters identified by setting the cut-off level at 4.0, a total of 19 known RpoE promoters were identified (Table 5, marked by asterisk). The majority of known promoters represent the inducible promoters that are expressed only under the support of positive regulatory factors.

**Table 5:** RpoE holoenzyme-binding sites on the *E*. *coli* K-12 genome.

No.	Type	Map	Left Gene Function	Left	D	RpoE	D	Right	Right Gene Function	Intensity
1	D	154236	fimbrial-like adhesin protein	***yadM***	<	***htrE***	<	***ecpD***		5.0
2	B	164658		***hrpB***	>		>	***mrcB***	glycosyl transferase and transpeptidase	4.3
3	B	202068		***lpxD***	>		>	***fabZ***	hydroxymyristol acyl carrier dehydratase	30.9
4	C	262270		***thrW***	>		<	***ykfI***		4.6
5	D	270562	CP4-6 prophage transcriptional regulator	***perR***	<	***insI***	<	***insH***		4.6
6	B	292358	CP4-6 prophage; conserved protein	***yagK***	<		<	***yagL***		4.7
7	D	302738	xanthine dehydrogenase 2Fe-2S subunit	***yagT***	<	***yagU***	<	***ykgJ***		5.3
8	A	319448	oxidoreductase with FAD-binding domain	*ykgC	<		>	***ykgD***	DNA-binding transcriptional regulator	4.0
9	C	323832		***ykgG***	>		<	***ykgH***		5.6
10	B	331456		***betT***	>		>	***yahA***	DNA-binding transcriptional regulator	4.9
11	d	343660		***yahK***	>	***yahL***	>	*yahM	predicted protein	17.8
12	A	345542	neutral amino-acid efflux system	***yahN***	<		>	***yahO***	predicted protein	4.0
13	B	477848	conserved inner membrane protein	***ylaB***	<		<	***ylaC***		4.5
14	D	543560	conserved protein	*ylbA	<	***allC***	<	***allD***		9.6
15	B	581644		***nohB***	>		>	***appY***	DLP12 DNA-binding transcriptional activator	11.5
16	B	629042		***ybdB***	>		>	***cstA***	carbon starvation protein	4.3
17	B	728638		***ybfA***	>		>	***rhsC***	rhsC element core protein RshC	5.6
18	D	732870		***ybfA***	>	***rhsC***	>	***ybfB***	predicted inner membrane protein	4.1
19	D	747240	predicted regulator	***abrB***	<	*ybgO	<	***ybgP***		9.3
20	A	753948	citrate synthase	***gltA***	<		>	***sdhC***	succinate dehydrogenase	4.7
21	B	773834		***ybgE***	>		>	***ybgC***	predicted acyl-CoA thioesterase	4.2
22	B	837732	conserved protein	***ybiI***	<		<	***ybiX***		4.1
23	D	838958	conserved protein	***ybiX***	<	***fiu***	<	***mcbA***		4.3
24	D	844530		***rlmF***	>	***ybiO***	<	***glnQ***		6.0
25	A	862768	pyruvate formate lyase activating enzyme	***ybiY***	<		>	***fsaA***	fructose-6-phosphate aldolase 1	4.4
26	B	887366	ncRNA	*rybB	<		<	***ybjL***		55.5
27	B	986332	outer membrane porin 1a (Ia;b;F)	***ompF***	<		<	***asnS***		5.0
28	D	992744		***pepN***	>	***ssuB***	<	***ssuC***		5.3
29	D	1045134	exopolysaccharide export protein	***gfcE***	<	***gfcD***	<	***gfcC***		5.1
30	C	1049830		***insB***	>		<	***cspH***		6.3
31	A	1050632	CspA-family stress protein	*cspH	<		>	***cspG***	DNA-binding transcriptional regulator	4.7
32	D	1088072	predicted glycosyl transferase	***pgaC***	<	***pgaB***	<	***pgaA***		4.8
33	B	1166772		***ndh***	>		>	***ycfJ***	predicted protein	4.0
34	C	1195868		***icd***	>		<	***ymfD***		6.4
35	B	1218154		***ycgG***	>		>	*ymgF	predicted protein	5.4
36	D	1259856		***ychH***	>	***ychM***	<	***prs***		5.3
37	D	1342036	lipoprotein	***osmB***	<	*yciT	<	***yciZ***		4.3
38	B	1349272	enoyl-[acyl-carrier-protein] reductaset	***fabI***	<		<	***ycjD***		5.6
39	B	1384744		***ycjF***	>		>	***tyrR***	DNA-binding transcriptional regulator	5.0
40	A	1389946	predicted hydrolase	***ycjY***	<		>	***ycjZ***	DNA-binding transcriptional regulator	17.2
41	B	1432738	Rac prophage transcriptional regulator	***ynaE***	<		<	***uspF***		5.6
42	D	1468142	KpLE2 phage-like IS repressor InsA	***insC***	<	***insI***	>	***ydbC***	NAD(P)-binding oxidoreductase	5.4
43	D	1500658		***tehB***	>	*ydcL	>	***ydcM***	predicted transposase	4.8
44	B	1518232		***yncB***	>		>	***mcbR***	DNA-binding transcriptional regulator	4.5
45	B	1542352	nitrate/nitrite transporter	***narU***	<		<	***yddJ***		4.9
46	B	1585730	fimbrial-like adhesin protein	***ydeQ***	<		<	***ydeR***		4.4
47	B	1588350	fimbrial-like adhesin protein	***ydeS***	<		<	***hipA***		4.5
48	A	1630638	predicted mannonate dehydrogenase	***ydfI***	<		>	*ydfK	Qin prophage transcriptional regulator	5.1
49	B	1638930	Qin prophage; predicted S lysis protein	***essQ***	<		<	***cspB***		7.0
50	A	1639660	Qin prophage; cold shock protein	***cspB***	<		>	*cspF	Qin prophage; cold shock protein	7.3
51	A	1676254	pyridine nucleotide transhydrogenase	***pntA***	<		>	***ydgH***	predicted protein	5.0
52	B	1751846	predicted oxidoreductase	***ydhV***	<		<	***ydhY***		6.6
53	A	1801758	threonyl-tRNA synthetase	***thrS***	<		>	***yniD***	predicted protein	10.1
54	D	1852370	predicted transporter	*ydjE	<	*ydjF	<	***ydjG***		4.4
55	B	1887966	acyl-CoA synthetase	***fadD***	<		<	***yeaY***		4.5
56	A	1923044	conserved protein	***yobA***	<		>	***holE***	DNA polymerase III	4.5
57	D	1938762	myristoyl-acyl carrier ACP acyltransferase	***lpxM***	<	*yebA	<	***znuA***		7.1
58	A	2060070	DNA-binding transcriptional dual regulator	***nac***	<		>	***asnV***	Asn tRNA	4.4
59	D	2103368	predicted acyl transferase	***wbbJ***	<	***wbbI***	<	***rfc***		4.2
60	D	2104870	conserved protein	***wbbI***	<	***rfc***	<	***glf***		5.7
61	D	2144358	uridine/cytidine kinase	***udk***	<	***yegE***	<	***alkA***		5.8
62	B	2196338		***metG***	>		>	***yehI***	conserved protein	8.6
63	D	2210570	conserved protein	***yehS***	<	***yehT***	<	***yehU***		5.2
64	D	2214150		***yohO***	>	***yehW***	<	***yehX***		4.0
65	D	2302258		***yojO***	>	***eco***	<	***mqo***		7.2
66	D	2369270		***arnD***	>	***arnT***	>	***arnE***	conserved protein	5.9
67	A	2405036	DNA-binding transcriptional repressor	***lrhA***	<		>	***yfbQ***	predicted aminotransferase	4.0
68	D	2441138	3-oxoacyl-[acyl-carrier-protein] synthase I	***fabB***	<	***mnmC***	<	***yfcL***		5.7
69	A	2459264	conserved protein	***yfcZ***	<		>	***fadL***	long-chain fatty acid OM transporter	5.3
70	D	2484966		***evgA***	>	***evgS***	<	***yfdE***		5.2
71	D	2490262	predicted oxalyl-CoA decarboxylase	*oxc	<	***frc***	<	***yfdX***		4.9
72	D	2492854	predicted protein	***yfdX***	<	*ypdI	<	***yfdY***		4.1
73	A	2507446	glucokinase	***glk***	<		>	***yfeO***	predicted ion channel protein	4.3
74	A	2535342	pyridoxal-pyridoxamine kinase	***pdxK***	<		>	*yfeK	predicted protein	7.5
75	D	2649866	fused transglycosylase/transpeptidase	***pbpC***	<	***yfhM***	>	***sseA***	3-mercaptopyruvate sulfurtransferase	6.1
76	A	2696642	conserved protein	***yfhB***	<		>	***yfhH***	DNA-binding transcriptional regulator	4.7
77	A	2708134	RNA polymerase sigma 24	***rpoE***	<		>	***nadB***	quinolinate synthase	6.3
78	B	2761570	CP4-57 prophage protein	***yfjK***	<		<	***yfjL***		5.6
79	B	2763272	CP4-57 prophage protein	***yfjL***	<		<	***yfjM***		3.2
80	D	2764730	CP4-57 prophage protein	***yfjM***	<	***rnlA***	>	***yfjO***	CP4-57 prophage protein	6.1
81	B	2773454		***yfjW***	>		>	***yfjX***	CP4-57 prophage antirestriction protein	6.1
82	C	2796040		***ygaP***	>		<	***stpA***		4.3
83	A	2812736	S-ribosylhomocysteinase	***luxS***	<		>	*micA	ncRNA	40.9
84	A	2874372	sulfate adenylyltransferase	***cysD***	<		>	*iap	aminopeptidase	4.2
85	D	2881556	predicted protein	***ygcK***	<	***ygcL***	<	***ygcB***		10.9
86	D	2894072	predicted flavoprotein	***ygcQ***	<	***ygcR***	<	***ygcS***		4.0
87	D	2903952	conserved protein	***ygcF***	<	*ygcG	<	***eno***		4.5
88	B	2985164		***yqeG***	>		>	***yqeH***	protein with bipartite regulator domain	4.0
89	A	3004270	DNA-binding transcriptional regulator	***ygeV***	<		>	*ygeW	conserved protein	4.5
90	D	3036652	ssDNA exonuclease	***recJ***	<	*dsbC	<	***xerD***		5.1
91	B	3237758		***alx***	>		>	***sstT***	sodium:serine/threonine symporter	23.4
92	D	3246540		***yqjA***	>	***yqjB***	>	***yqjC***	conserved protein	4.4
93	A	3276944	DNA-binding transcriptional dual regulator	***agaR***	<		>	***kbaZ***	tagatose 6-phosphate aldolase 1	4.2
94	B	3375554	stringent starvation protein A	***sspA***	<		<	***rpsI***		4.7
95	B	3387142	p-hydroxybenzoic acid efflux system	***aaeA***	<		<	***aaeX***		4.9
96	C	3528652		***hslO***	>		<	***yhgE***		4.1
97	A	3559934	thiosulfate:cyanide sulfurtransferase	***glpE***	<		>	***glpD***	sn-glycerol-3-phosphate dehydrogenase	4.0
98	B	3576850	DNA-binding transcriptional repressor	***gntR***	<		<	***yhhW***		4.9
99	A	3579162	ncRNA	***ryhB***	<		>	***yhhY***	predicted acetyltransferase	6.5
100	B	3599044	RNA polymerase sigma 32	*rpoH	<		<	***ftsX***		37.4
101	C	3661646		***mdtF***	>		<	***gadW***		6.7
102	D	3666862	glutamate decarboxylase A	***gadA***	<	***yhjA***	>	***treF***	cytoplasmic trehalase	6.6
103	B	3720058		***insK***	>		>	***sokA***	ncRNA	7.3
104	B	3739660	4Fe-4S ferredoxin-type hydrogenase	***ysaA***	<		<	***yiaJ***		4.2
105	D	3764054	predicted glutathione S-transferase	***yibF***	<	***rhsA***	>	***yibA***	lyase containing HEAT-repeat	4.0
106	B	3779166		***lldD***	>		>	***yibK***	predicted rRNA methylase	10.8
107	B	3828470		***yicE***	>		>	***yicH***	conserved protein	4.2
108	D	3829430		***yicE***	>	***yicH***	<	***yicI***		8.2
109	D	3862372		***yidL***	>	***yidP***	<	***yidE***		4.3
110	B	3906432	phosphate transporter subunit	***pstB***	<		<	***pstA***		5.2
111	D	3955270		***ilvA***	>	***ilvY***	>	***ilvC***	ketol-acid reductoisomerase	4.1
112	A	3958552	peptidyl-prolyl cis-trans isomerase C	***ppiC***	<		>	***rep***	DNA helicase/ssDNA-dependent ATPase	4.8
113	B	3978772		***rffM***	>		>	***yifK***	predicted transporter	4.0
114	B	4068538	predicted aldose-1-epimerase	***yihR***	<		<	***yihS***		10.7
115	B	4173964		***thrT***	>		>	***tufB***	protein chain elongation factor EF-Tu	5.7
116	B	4187734		***rpoC***	>		>	***yjaZ***	heat shock protein	4.0
117	A	4212172	predicted acetyltransferase	***yjaB***	<		>	***metA***	homoserine O-transsuccinylase	4.3
118	B	4249366		***malM***	>		>	***ubiC***	chorismate pyruvate lyase	5.7
119	B	4364770	C4-dicarboxylate antiporter	***dcuA***	<		<	***aspA***		5.2
120	A	4368630	predicted transporter	***yjeH***	<		>	***groS***	Cpn10 chaperonin GroES	4.3
121	A	4380530	anaerobic fumarate reductase	***frdA***	<		>	***poxA***	predicted lysyl-tRNA synthetase	4.0
122	B	4427854		***fklB***	>		>	***cycA***	D-alanine/D-serine/glycine transporter	8.0
123	D	4486746		***yjgQ***	>	***yjgR***	<	***idnR***		9.4
124	B	4530030	KpLE2 phage endoglucanase	***sgcX***	<		<	***yjhP***		5.2
125	B	4533162	conserved protein	***yjhX***	<		<	***yjhS***		4.0
126	A	4538964	N-acetylnuraminic acid outer membrane channel protein	***nanC***	<		>	***fimB***	tyrosine recombinase/*fimA* regulator	6.4
127	B	4638610		***yjjY***	>		>	*yjtD	predicted rRNA methyltransferase	4.6

Genomic SELEX was performed for search of the binding sites of RNAP RpoE holoenzyme. By setting the cut-off level of 4.0, a total of 126 binding sites were identified (see Fig 4 for SELEX pattern), which are aligned along the map of *E*. *coli* K12 genome. A total of 84 sites are located within intergenic spacers: 29 wihin type-A spacers (shown under orange background); and 48 within type-B spacers (shown under green background). The constitutive promoters of RpoE were predicted based on the adjacent genes [note that only the genes next to the RpoE holoenzyme-binding sites are shown] and the gene orientation (shown by arrows in the column of transcription direction). A total of 42 RpoE holoenzyme-binding sites are located inside open reading frames as indicated by the gene symbols shown in RpoE column.

*The genes listed in RegulonDB as the regulated targets of RpoE.

Genomic SELEX analysis identified minimum 77 and maximum 106 for the RpoE constitutive promoters. The highest peak (55-fold intensity) was detected in the promoter region of *rybB*, which encodes a small regulatory RNA for expression control of some outer membrane proteins. The *rybB* promoter is known to be regulated by RpoE (Fig 4) [67]. The second highest peak was located on the *luxS-micA* intergenic region. The *micA* gene again encodes a small regulatory RNA that regulates expression of many genes including outer membrane proteins [68,69]. The *micA* promoter is also established under the control of RpoE. These sRNAs control the repair of damages in the outer membrane that took place in response to envelope stress [70,71]. High-intensity peaks were detected on some other known RpoE-dependent promoters such as *rpoH*, *pgrR* and *ycjY* [72,73]. Among the total of 136 binding sites of RpoE holoenzyme, 36 overlaps with that of RpoH holoenzyme. Most of these overlapping sites are related to the genes that are expressed under envelope stresss or heat-shock stress.

### The intracellular levels of all seven sigma factors in *E*. *coli* K-12 W3110

In this study, we determined the constitutive promoters for the four minor sigma factors, RpoS, RpoH, RpoF and RpoE, from *E*. *coli* K-12 W3110. These promoters are recognized by the RNAP holoenzyme containing each sigma in the absence of other supporting factors. Using the mixed reconstitution *in vitro* of RNAP holoenzyme in the presence of all seven sigma factors, we estimated the binding affinity of each sigma to the common core enzyme, the order being RpoD (highest) > RpoN > RpoF > RpoH > FecI > RpoE > RpoS (lowest) [74]. Once we get the knowledge of intracellular concentrations of these sigma factors, it should be possible to predict the expression levels of the regulatory target genes and operons under the control of the constitutive promoters of each sigma factor. Including these four minor sigma factors, we then determined the intracellular concentrations of all seven sigma subunits. For this purpose, antibodies were made against each of the purified sigma factors that were also used for SELEX screening.

**Fig 5:**
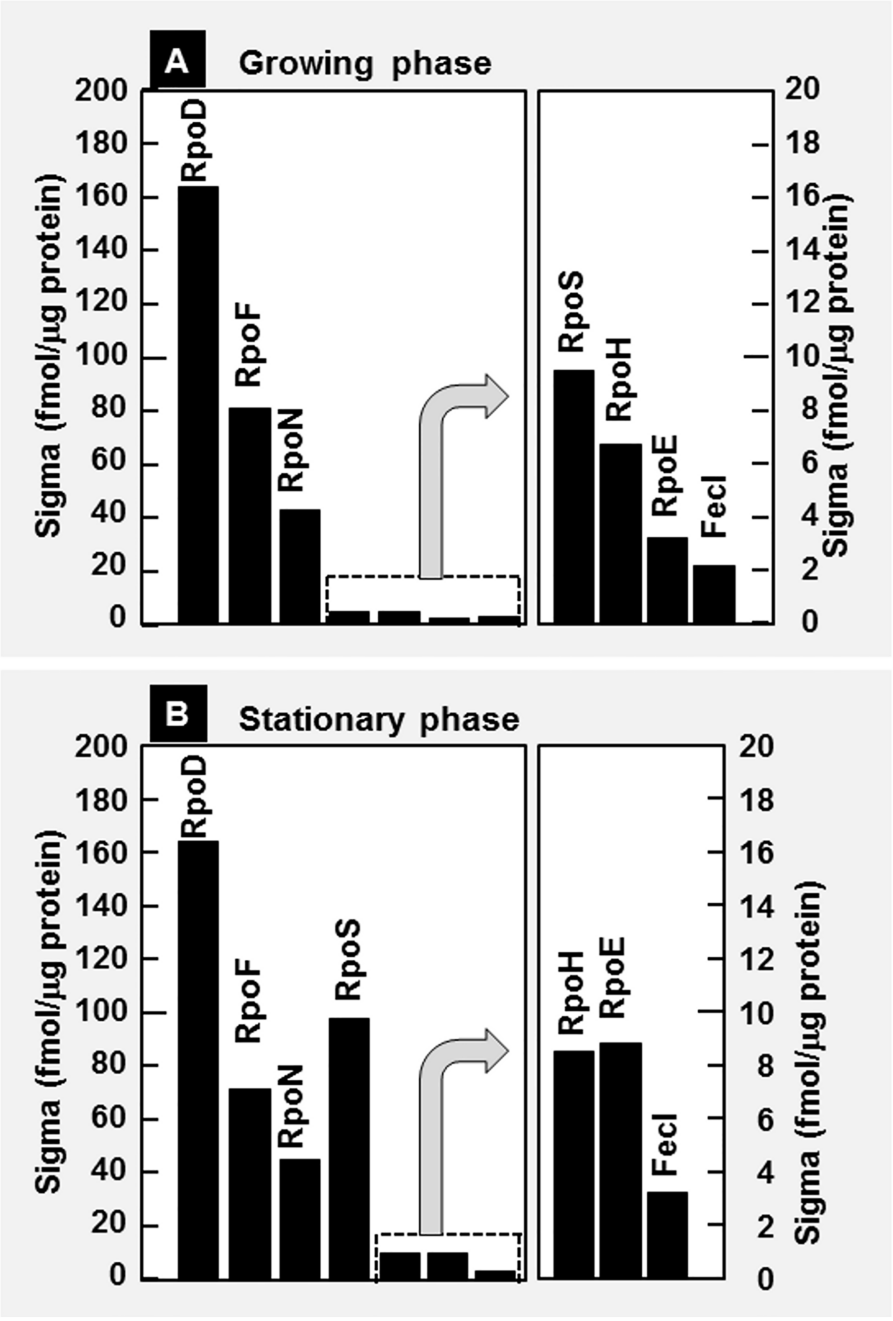
Intracellular concentrations of seven sigma factors in *E*. *coli* K-12 W3110 type-A strain. *E*. *coli* K-12 W3110 type-A strain was grown in LB medium at 37°C with shaking. Cells were harvested at various times and cell lysates were subjected to the quantitative immuno-blot analysis of all seven sigma factors as described in Materials and Methods. [A] The sigma levels at exponential growth phase; [B] the sigma levels in the stationary phase.

*E*. *coli* K-12 W3110 type-A was cultivated with shaking at 37°C in LB medium, and the whole cell lysates were prepared in both exponential growing phase and the stationary phase. By using the quantitative immuno-blotting method and the purified sigma proteins as the reference controls, we measured the concentrations of all seven sigma subunits. The measurement was carried out for two independent cultures, and the immuno-blot analysis was repeated for all the samples. The intracellular concentration of RpoD sigma is maintained at a constant level (on average, 160 fmol/μg total protein) throughout the transition from the exponential growth phase to the stationary phase (Fig 5) in good agreement with the previous estimation [1,75]. Based on the relative level of RNAP core enzyme subunits, the number of RpoD sigma was estimated to be 800 molecules per genome [note that the number of RNAP core enzyme is 2,000 molecules per genome]. The level of RpoD sigma in *E*. *coli* K-12 MG1655 was about half the level of W3110 (data not shown). The second abundant sigma was RpoF (70 fmol/μg protein at log phase and 80 fmol/μg protein at stationary phase) (Fig 5), but the unneccesary RpoF is stored in an inactive form by forming complexes with the anti-RpoF sigma, FlgM [76]. RpoN is also always present at a constant level (approximately 40 fmol/μg protein) in both log and stationary phases (Fig 5). The levels of other four sigma, RpoS, RpoH, RpoE and FecI, are very low during the steady-state growth (Fig 5A), but upon entry into the stationary phase, the level of RpoS increased markedly up to the level (about 100 fmol/μg protein) higher than other five minor sigma factors (Fig 5B). Taking the intracellular concentrations and the binding affinity of sigma to RNAP core enzyme as noted above, we are now able to estimate the level of each RNAP holoenzyme. Noteworthy is that the total number of all seven sigma factors is approximately as many as that of the core enzyme, but the RNAP involved in the transcription cycle or the elongation of RNA chains is considered to lack sigma subunit, the RNAP not involved in transcription should be stored as the holoenzyme forms.

## Discussion

Seven species of the sigma subunit exist in *E*. *coli* K-12, the widely used model *E*. *coli* strain. Here we identified the whole set of constitutive promoters for four minor sigma factors, RpoS, RpoH, RpoF and RpoE, by using the Genomic SELEX system. Up to the present time, the binding sites of RNAP and TF have been identified *in vivo* using the high-throughput systems such as ChIP-chip, ChIP-seq and RNA-seq systems. Even using these modern techniques, however, it is in principle impossible to obtain the whole set of binding sites for both RNAP and TFs because of the competition with other DNA-binding proteins in binding to DNA targets [8,9,24] [note that *E*. *coli* contains more than 500 DNA-binding proteins [77], and because the binding of RNAP and TFs often depends on the supporting factors for binding to targets [8,9,25].

The computational approaches *in silico* have also been employed to identify the target binding sequences of RNAP and TFs, relying on the consensus sequences predicted based on the known target sequences listed in the databases such as RegulonDB [20,21] and EcoCyc [22,23] (Table 2) (Details of the promoter list and the evidence are in Supplemental Information: S1 Table for RpoS; S2 Table for RpoH; S3 Table for RpoF; and S4 Table for RpoE). In particular, more than 80% of RpoE-dependent promoters were predicted *in silico* (Table 5; and S4 Table). The consensus sequences, however, often include the inaccurate non-target sequences due to the lack of experiments for confirmation or some regulators recognize wide-varieties of the binding sequences [8,9,27,78]. Another serious problem associated with *in vivo* approaches is the difference in genetic background of *E*. *coli* strains used. Up to the present, the complete genome sequence has been determined for more than 1,000 different *E*. *coli* strains, allowing the prediction of about 3,000 core genes for all strains but at least one third of the total genes on the *E*. *coli* genome are different among the hitherto sequenced *E*. *coli* genome [79]. The difference in genetic background exists even in the RNAP and TF genes and between not only different strains but also different stocks of the same *E*. *coli* strain. For instance, the difference in the gene encoding the stationary-phase sigma RpoS was first identified between laboratory stocks of a single and the same *E*. *coli* K-12 W3110 strain [80]. The widely used databases such as RegulonDB [20,21] and EcoCyc [22,23] include huge collections of useful data of transcription *in vivo*, but care should be taken to use these data for theoretical prediction of transcription regulation, in particular, as to the bacterial strains and culture conditions used in each experiment.

In this study, we performed the SELEX screening for the constitutive promoters that are recognized *in vitro* by four minor sigma factors, RpoS, RpoH, RpoF and RpoE, but in the absence of repressors, activators and other DNA-binding proteins. It should be noted that all the proteins and promoters used in this study are prepared from a single and the same *E*. *coli* K-12 W3110. Here we also determined the intracellular concentrations of all seven sigma factors in both growing and stationary-phase cells of *E*. *coli* K-12 W3110. These data altogether will be used for our ultimate purpose of the prediction of genome expression under a given condition. The list of constitutive promoters for the minor sigma factors will be deposited into TEC database (Transcriptional profile of *Escherichia coli* database: https://shigen.nig.ac.jp/ecoli/tec/top/) [9].

## Materials and methods

### Bacterial strains and plasmids

*E*. *coli* K12 W3350 type-A containing the full-set of seven sigma factors [80] was used for purification of RNA polymerase and the template DNA for Genomic SELEX screening of RpoS, RpoH, RpoF and RpoE promoters. *E*. *coli* BL21(DE3) was used for the expression and purification of sigma and core enzyme subunit proteins. Expression plasmids for the core enzyme subunits (pRpoA, pRpoB and pRpoC) and all seven sigma subunits (pRpoD, pRpoN, pRpoS, pRpoH, pRpoF, pRpoE and pFecI) were constructed by ligating the respective coding sequences, which were prepared by PCR amplification of the *E*. *coli* K12 W3350 type-A genome DNA as template, into pET21 expression vector essentially according to the standard procedure used for expression of all sigma and all transcription factors in this laboratory [74,81].

### Purification of core RNA polymerase

RNAP was purified from log-phase cells of *E*. *coli* K-12 W3350 by the standard procedure [29]. Separation of the native core from holoenzymes was performed by passing the purified RNAP through P11-phosphocellulose column in the presence of 50% glycerol. To remove trace amounts of the core enzyme-associated sigma factors, the purified RNAP in the storage buffer containing 50% glycerol was dialyzed against the same buffer but containing 5% glycerol and fractionated by P11-phosphocellulose column chromatography in the presence of 5% glycerol. The level of remaining sigma factors was less than 0.1%, if any, as checked of SDS-PAGE gels by both protein-staining with a silver reagent and immuno-staining with antibodies against each of seven sigma factors.

### Purification of core and sigma subunits

The core enzyme subunits (RpoA, RpoB, RpoC and RpoZ) were expressed using the respective expression plasmids and purified by two cycles of column chromatography through DEAE (DE52) and P11-phosphocellulose [29]. Sigma subunits were expressed and purified by ion-exchange column chromatography through DE52 and P11 followed by Sephacryl S-300 gel filtration column. The purified sigma and core subunit proteins were more than 99% pure as judged by both protein-staining and immuno-staining of SDS-PAGE gels.

### Preparation of antibodies

Antibodies against sigma factors and core enzyme subunits were produced in rabbits by injecting purified sigma proteins [75,76]. Antibodies against each RNA polymerase protein were produced in two rabbits, and after examination of antibody activity using immune-blot analysis, the batch of higher activity was used in this study. Anti-RpoD, anti-RpoS, anti-RpoN, anti-RpoH, anti-RpoF, anti-RpoE, anti-FecI and anti-RpoC used in this study did not cross-react with each other. These antibodies were produced in the Nippon Institute for Biological Science (One, Tokyo) and the Animal Laboratory of Mitsubishi Chemical Medience Co. (Uto, Kumamoto, Japan).

### Genomic SELEX screening of RNA polymerase holoenzyme binding sequences

The Genomic SELEX screening was carried out under the standard procedure [26,27]. This method was developed by improvement of the original SELEX methods [30–32]. A mixture of DNA fragments of the *E*. *coli* K-12 W3110 genome was prepared after sonication of purified genome DNA, and cloned into a multi-copy plasmid pBR322 at EcoRV site. In each SELEX screening, the DNA mixture was regenerated by PCR using a pair of primers with the flanking sequences of pBR322 EcoRV. For SELEX screening, 5 pmol of the mixture of DNA fragments and 10 pmol of RNA polymerase holoenzyme were mixed in a binding buffer (10 mM Tris-HCl, pH 7.8 at 4°C, 3 mM magnesium acetate, 150 mM NaCl, and 1.25 mg/ml bovine serum albumin) and incubated for 30 min at 37°C. The DNA-RNA polymerase mixture was treated with anti-RpoC antibody and DNA fragments recovered from the complexes were PCR-amplified and subjected to next cycle of SELEX for enrichment of RNA polymerase-bound DNA fragments.

For SELEX-chip analysis, DNA samples were isolated from the DNA-protein complexes at the final state of SELEX, PCR-amplified and labeled with Cy5 while the original DNA library was labeled with Cy3. The fluorescent labeled DNA mixtures were hybridized to a DNA microarray consisting of 43,450 species of 60 b-long DNA probe, which are designed to cover the entire *E*. *coli* K-12 MG1655 genome at 105 bp interval (Oxford Gene Technology, Oxford, UK) [14]. The fluorescent intensity of test sample at each probe was normalized with that of the corresponding peak of original library. After normalization of each pattern, the Cy5/Cy3 ratio was measured and plotted along the *E*. *coli* K-12 MG1655 genome. The gene organization is almost identical between two well-characterized *E*. *coli* K-12 strains except for a long-range inversion between the *rrnD* and *rrnE* operons.

### Immuno-blot analysis for determination of sigma levels

For the measurement of sigma factors in *E*. *coli* K-12 W3110, a quantitative Western blot analysis was employed with the anti-sigma antibodies as employed in the previous studies [66,75,76]. In brief, cell lysates were treated with a SDS (sodium dodecyl sulfate) sample buffer (50 mM Tris-HCl, pH 6.8, 2% SDS, 1% 2-mercaptoethanol, 10% glycerol, and 0.025% bromophenol blue) and separated on SDS–7.5 or 10% polyacrylamide gels. Proteins in gels were directly electro-blotted onto polyvinylidene difluoride membranes (Nippon Genetics). Blots were blocked overnight at 48C in 3% BSA in PBS (phosphate-buffered saline), probed with the polyclonal antibodies against each sigma factor, washed with 0.5% Tween 20 in PBS, and incubated with goat anti-rabbit immunoglobulin G conjugated with hydroxyperoxidase (Cappel). The blots were developed with 3,3’-diaminobenzidine tetrahydrochloride (Dojindo). Staining intensity was measured with a PDI image analyzer system equipped with a white light scanner. The standard curve for the calculation of each sigma level was prepared from the immuno-blot patterns of increasing concentrations of each sigma factor. Under the standard Western-blot conditions herein employed, the linearity was detected over a 10-fold range at least between 2 and 20 ng sigma proteins. The determination of test sigma proteins subunits was first performed using several different volumes of the cell lysates. Using the optimum volumes of cell lysates to give the sigma concentrations within the linear range of standard curves, we finally repeated the determination of individual sigma factors.

## Supporting information

S1 Table RpoS-dependent promoters listed in RegulonDB. Promoters listed in RegulonDB are classified into those not identified as the constitutive promoters (A) and the constitutive promoters identified by SELEX screening (B). Evidence for each promoter are as described in RegulonDB (see Table 5): (Group-A) Promoters were experimentally identified by using HTTIM (high-throughput transcription initiation mapping), TIM (transcription initiation mapping), FP (footprinting), or IDA (inferred by direct promoter assay; (Class-B) Promoters were predicted based on AIPP (automated inference of promoter position), ICWHO (inferred computationally without human oversight), HIPP (human inference of promoter position), NTAS (non-traceable author statement), TASES (traceable author statement to experimental support), TAS (traceable author statement), IMP (inferred from mutant) or IEP (inferred from expression pattern).
(PDF)

S2 Table RpoH-dependent promoters listed in RegulonDB. Promoters listed in RegulonDB are classified into those not identified as the constitutive promoters (A) and the constitutive promoters identified by SELEX screening (B). Evidence for each promoter are as described in S1 Table.
(PDF)

S3 Table RpoF-dependent promoters listed in RegulonDB. Promoters listed in RegulonDB are classified into those not identified as the constitutive promoters (A) and the constitutive promoters identified by SELEX screening (B). Evidence for each promoter are as described in S1 Table.
(PDF)

S4 Table RpoS-dependent promoters listed in RegulonDB. Promoters listed in RegulonDB are classified into those not identified as the constitutive promoters (A) and the constitutive promoters identified by SELEX screening (B). Evidence for each promoter are as described in S1 Table.
(PDF)
