# Supplementary material for: The whole set of the constitutive promoters recognized by four minor sigma subunits of Escherichia coli RNA polymerase
Source: PLoS One. 2017 Jun 30;12(6):e0179181. doi: 10.1371/journal.pone.0179181 (PMC5493296; doi:10.1371/journal.pone.0179181)
Supplement: S1 Table — Promoters listed in RegulonDB are classified into those not identified as the constitutive promoters (A) and the constitutive promoters identified by SELEX screening (B). Evidence for each promoter are as described in RegulonDB (see Table 5): (Group-A) Promoters were experimentally identified by using HTTIM (high-throughput transcription initiation mapping), TIM (transcription initiation mapping), FP (footprinting), or IDA (inferred by direct promoter assay; (Class-B) Promoters were predicted based on AIPP (automated inference of promoter position), ICWHO (inferred computationally without human oversight), HIPP (human inference of promoter position), NTAS (non-traceable author statement), TASES (traceable author statement to experimental support), TAS (traceable author statement), IMP (inferred from mutant) or IEP (inferred from expression pattern). (PDF) [file pone.0179181.s001.pdf]

S1 Table
RpoS promoters (RegulonDB)

[A] Promoters not identified as the constitutive promoters

Promoter	SELEX	Direction	Genome position	Evidence
ldcCp	-	forward	0	IMPIW, TASIW
fliYp	-	reverse	0	IEPIW, IMPIW
nhaAp2	-	forward	17317	TIMIS
yabIp	-	forward	71271	HTTIMIS, TIMIS
ftsQp2	-	forward	102742	HIPPIW, TIMIS
ftsQp1	-	forward	102867	HIPPIW, TIMIS
yadEp4	-	forward	145053	ICWHOIW
yadVp2	-	reverse	156224	HIPPIW, TIMIS
pcnBp4	-	reverse	159391	TIMIS
hrpBp2	-	forward	162075	ICWHOIW
dkgBp	-	forward	229098	AIPPIW
insN-1p	-	forward	269450	HTTIMIS
adrAp	-	forward	402911	HIPPIW, TIMIS
bolAp1	-	forward	453658	AIPPIW, CV(FP/TIM), FPIS, TIMIS
ybgAp2	-	forward	738143	TIMIS
ybgAp1	-	forward	738203	TIMIS
sucAp	-	forward	757809	TIMIS
ybhBp4	-	reverse	807153	ICWHOIW
rhlEp5	-	forward	830006	ICWHOIW
ybjPp	-	reverse	903746	TIMIS
poxBp	-	reverse	910299	HTTIMIS, TIMIS
ompFp	-	reverse	986315	CV(FP/TIM), FPIS, HIPPIW
cbpAp2	-	reverse	1063051	CV(FP/TIM), FPIS, HIPPIW, TIMIS
wrbAp	-	reverse	1066981	HTTIMIS
csgDp2	-	reverse	1102558	TIMIS
csgDp1	-	reverse	1102567	TIMIS
csgBp	-	forward	1103082	CV(FP/TIM), FPIS, TIMIS
msyBp	-	reverse	1113436	TIMIS
treAp	-	reverse	1246648	HIPPIW, IMPIW, TIMIS
rssAp	-	forward	1288329	IMPIW, TIMIS
rssBp	-	forward	1289443	HIPPIW, TIMIS
adhEp2	-	reverse	1297532	HIPPIW, TIMIS
adhEp1	-	reverse	1297636	IMPIW, TIMIS
ychEp8	-	forward	1297675	ICWHOIW, HIPPIW
yciGp	-	reverse	1314116	AIPPIW, HIPPIW

acnAp1	-	forward	1333448	HIPPIW, TIMIS
yciTp	-	reverse	1342534	HIPPIW
gmrp	-	reverse	1344867	HIPPIW, TIMIS
puuCp	-	forward	1360752	HTTIMIS
ydbDp	-	forward	1473090	AIPPIW, IEPIW
ydcSp1	-	forward	1509623	HIPPIW
ansPp1	-	reverse	1524035	TIMIS
ansPp2	-	reverse	1524044	TIMIS
narUp2	-	reverse	1542187	HIPPIW
yddGp	-	reverse	1545229	TIMIS
srap	-	reverse	1554070	TIMIS
osmCp2	-	forward	1554630	HIPPIW, TIMIS
ddpXp2	-	reverse	1561225	HIPPIW
ddpXp3	-	reverse	1561273	HIPPIW
lsrAp	-	forward	1599484	AIPPIW
tamp	-	forward	1605245	HTTIMIS, IEPIW
asrp	-	forward	1669351	TIMIS
fumCp	-	reverse	1684733	TIMIS
sodCp	-	reverse	1722703	HTTIMIS, TIMIS
ydhYp	-	reverse	1752666	TIMIS
pfkBp2	-	forward	1804347	TIMIS
pfkBp1	-	forward	1804375	HIPPIW, TIMIS
katEp	-	forward	1811838	IMPIW, TIMIS
astCp3	-	reverse	1830063	CV(FP/TIM), FPIS, HIPPIW, TIMIS
xthAp	-	forward	1830423	HIPPIW, TIMIS
yobBp8	-	forward	1923233	ICWHOIW
lpxMp4	-	reverse	1938252	ICWHOIW
znuAp	-	reverse	1940685	AIPPIW
otsAp	-	reverse	1979675	HIPPIW, TIMIS
otsBp2	-	reverse	1980466	TIMIS
otsBp	-	reverse	1980489	HIPPIW, TIMIS
araFp2	-	reverse	1984172	HTTIMIS
hchAp2	-	forward	2033654	TIMIS
alkAp	-	reverse	2145583	FPIS
mdtAp	-	forward	2152003	AIPPIW
yegSp8	-	forward	2166707	ICWHOIW
fbaBp	-	reverse	2176689	AIPPIW, TIMIS
osmFp	-	reverse	2217545	TIMIS
yeiLp	-	forward	2253312	HIPPIW, TIMIS
adap	-	reverse	2308449	TIMIS
fadLp	-	forward	2459227	HIPPIW, TIMIS

yffOp	-	forward	2560075	HTTIMIS
talAp2	-	forward	2576592	TIMIS
talAp1	-	forward	2576610	TIMIS
tktBp	-	forward	2577330	HIPPIW
ppkp	-	forward	2620999	HTTIMIS
hmpAp	-	forward	2683819	CV(FP/TIM), FPIS, TIMIS
csiDp	-	forward	2786949	TIMIS
gabDp2	-	forward	2788934	HIPPIW, TIMIS
ygaUp4	-	reverse	2794836	ICWHOIW
luxSp1	-	reverse	2813087	HIPPIW, TIMIS
csrAp3	-	reverse	2817295	HIPPIW, TIMIS
mutSp	-	forward	2855041	TIMIS
ftsBp	-	reverse	2870923	AIPPIW
ssrSp2	-	forward	3053781	AIPPIW, TIMIS
yggEp1	-	reverse	3066148	TIMIS
yggEp2	-	reverse	3066207	TIMIS
speBp	-	reverse	3081968	HIPPIW, TIMIS
speCp	-	reverse	3107197	AIPPIW, HIPPIW
dkgAp	-	forward	3154593	HTTIMIS
tolCp4	-	forward	3176096	AIPPIW, TIMIS
insC-5p	-	forward	3184062	HTTIMIS
glgSp2	-	reverse	3190024	TIMIS
glgSp1	-	reverse	3190034	HIPPIW, TIMIS
patAp	-	forward	3217480	AIPPIW, TIMIS
yhaJp7	-	reverse	3252283	ICWHOIW
dacBp5	-	forward	3326879	ICWHOIW
slyDp7	-	reverse	3476550	ICWHOIW
ficp	-	reverse	3489670	HIPPIW
hofMp	-	reverse	3520950	AIPPIW
glgCp3	-	reverse	3567594	TIMIS
panZp7	-	forward	3595981	ICWHOIW
yhhTp4	-	forward	3609864	ICWHOIW
hdeAp	-	reverse	3654814	HIPPIW, TIMIS
gadXp	-	reverse	3663862	HIPPIW, TIMIS
gadAp	-	reverse	3665630	HIPPIW, TIMIS
treFp	-	forward	3667574	HTTIMIS
yhjGp	-	reverse	3676435	HTTIMIS
glySp4	-	reverse	3722518	ICWHOIW
aldBp	-	reverse	3754558	TIMIS
gpmMp2	-	forward	3783210	HTTIMIS
dnaNp2	-	reverse	3880613	HIPPIW, TIMIS

dnaNp1	-	reverse	3880756	HIPPIW, TIMIS
mnmGp	-	reverse	3923697	TIMIS
hdfRp	-	reverse	3946044	IHBCEIW
yigMp3	-	forward	4009073	ICWHOIW
yihGp	-	reverse	4044675	TIMIS
cpxRp	-	reverse	4103709	HIPPIW
glpXp2	-	reverse	4113629	ICWHOIW
rraAp	-	reverse	4117381	HTTIMIS, TIMIS
oxyRp	-	forward	4156480	HIPPIW, TIMIS
rsdp1	-	reverse	4194977	HIPPIW, TIMIS
yjcCp	-	forward	4273441	HTTIMIS
proPp2	-	forward	4328430	HIPPIW, TIMIS
ecnBp	-	forward	4374532	TIMIS
blcp	-	reverse	4375768	TIMIS
ytfKp	-	forward	4437534	TIMIS
mplp1	-	forward	4453751	HIPPIW
yjgBp10	-	reverse	4494257	ICWHOIW
iraDp2	-	forward	4554879	HIPPIW, TIMIS
yjiYp	-	reverse	4589390	TIMIS
yjjKp11	-	reverse	4628589	ICWHOIW

[B] Promoters identified as the constitutive promoters by SELEX

artPp1	154.2	reverse	903038	TIMIS
artPp2	154.2	reverse	903059	TIMIS
artPp3	154.2	reverse	903066	TIMIS
evgAp1	126.6	forward	2481663	TIMIS
dsrBp5	88.4	reverse	2022867	ICWHOIW
yciZp	48.2	reverse	1342662	TIMIS
uspBp	22.7	reverse	3637871	HIPPIW, TIMIS
puuAp	21.8	reverse	1358992	AIPPIW, HIPPIW, TIMIS
puuDp	21.8	forward	1359076	TIMIS
ybiIp6	19.6	reverse	837707	ICWHOIW
dhaKp	15.7	reverse	1250167	AIPPIW
dhaRp	15.7	forward	1250226	AIPPIW
gadBp	14.9	reverse	1570096	HIPPIW, TIMIS
ycgZp	14.2	forward	1214975	HIPPIW, TIMIS
yhiMp	13.3	forward	3632766	AIPPIW
appYp2	12.9	forward	582879	HTTIMIS
crrpI	9.2	forward	2533632	TIMIS
gadEp1	9.1	forward	3656265	IEPIW, TIMIS

gadEp	9.1	forward	3656368	TIMIS
ihfAp4	8.1	reverse	1793745	HIPPIW, TIMIS
yiaGp	6.1	forward	3717454	TIMIS
pykFp2	4.2	forward	1753624	HTTIMIS
ihfBp	3.6	forward	962972	HIPPIW, TIMIS
osmBp1	3.4	reverse	1341500	TIMIS
cfap1	2.9	forward	1739225	HIPPIW, TIMIS
cfap2	2.9	forward	1739403	HIPPIW, TIMIS
gadYp	2.2	forward	3662887	AIPPIW, HIPPIW, TIMIS
