# Supplementary material for: The whole set of the constitutive promoters recognized by four minor sigma subunits of Escherichia coli RNA polymerase
Source: PLoS One. 2017 Jun 30;12(6):e0179181. doi: 10.1371/journal.pone.0179181 (PMC5493296; doi:10.1371/journal.pone.0179181)
Supplement: S2 Table — Promoters listed in RegulonDB are classified into those not identified as the constitutive promoters (A) and the constitutive promoters identified by SELEX screening (B). Evidence for each promoter are as described in S1 Table. (PDF) [file pone.0179181.s002.pdf]

S2 Table
RpoH promoter (RegulonDB)

[A] Promoters not identified as the constitutive promoters

Promoter	SELEX	Direction	Genome position	Evidence
yjiTp	-	forward	0	IDAIS, IEPIW
yafUp	-	reverse	0	HIPPIW, IDAIS, TIMIS
yehRp	-	forward	0	IDAIS, IEPIW
ydeOp	-	reverse	0	IDAIS, IEPIW
yiaAp	-	reverse	0	IDAIS, IEPIW
yccEp	-	forward	0	IDAIS, IEPIW
yrdAp	-	forward	0	IDAIS, IEPIW
yjhIp	-	reverse	0	IDAIS, IEPIW
yrfGp	-	forward	0	IDAIS, IEPIW
casDp	-	reverse	0	IDAIS, IEPIW
osmFp2	-	reverse	0	IDAIS, IEPIW
hfqp1	-	forward	0	IDAIS, IEPIW
htgAp1	-	forward	10644	HIPPIW, TIMIS
yaaWp3	-	reverse	11542	ICWHOIW, TIMIS
ileSp3	-	forward	22229	AIPPIW, TIMIS
caiEp5	-	reverse	35440	ICWHOIW
kefFp2	-	forward	47080	ICWHOIW
hepAp2	-	reverse	63588	HIPPIW, IDAIS, TIMIS
fruRp8	-	forward	87947	ICWHOIW
yadDp5	-	forward	146957	ICWHOIW
yadDp4	-	forward	146959	ICWHOIW
rrsHp1	-	forward	223485	HIPPIW, ICWHOIW
yafDp	-	forward	231064	AIPPIW, IDAIS, TIMIS
yafCp6	-	reverse	231083	ICWHOIW
ivyp5	-	forward	240329	ICWHOIW
proBp11	-	forward	259527	ICWHOIW
mmuPp6	-	forward	274430	ICWHOIW
yagGp8	-	forward	284577	ICWHOIW
ykgJp3	-	reverse	303486	ICWHOIW
ykgEp10	-	forward	320761	ICWHOIW
betAp5	-	reverse	326485	ICWHOIW
yahNp5	-	reverse	345659	ICWHOIW
yahNp6	-	reverse	345660	ICWHOIW
mhpTp	-	forward	374610	AIPPIW
yaiOp5	-	reverse	380197	ICWHOIW

yaiSp9	-	reverse	383932	ICWHOIW
insFp5	-	reverse	391843	ICWHOIW
yajDp6	-	forward	429693	ICWHOIW
ribEp	-	forward	433728	AIPPIW, TIMIS
yajLp2	-	reverse	442972	ICWHOIW
ampGp4	-	reverse	452910	ICWHOIW
clpPp3	-	forward	455829	IDAIS, IEPIW, TIMIS
lonp	-	forward	458039	HIPPIW, IDAIS, IEPIW
ppiDp1	-	forward	461062	HIPPIW, TIMIS
htpGp1	-	forward	494299	HIPPIW, IDAIS, TIMIS
htpGp2	-	forward	494308	HIPPIW, IDAIS, TIMIS
fsrp8	-	reverse	503991	ICWHOIW
fsrp9	-	reverse	504079	ICWHOIW
ybbNp1	-	reverse	517714	AIPPIW, IDAIS, IEPIW
ybbNp2	-	reverse	517792	AIPPIW
ybcJp5	-	reverse	556229	ICWHOIW
ybcNp6	-	forward	571537	ICWHOIW
quuDp3	-	forward	572997	ICWHOIW
ybdJp3	-	reverse	605634	ICWHOIW
entDp1	-	reverse	609477	ICWHOIW
ybeFp6	-	reverse	660733	ICWHOIW
rlpAp5	-	reverse	664445	ICWHOIW
ybeQp10	-	reverse	675847	ICWHOIW
ybeUp5	-	forward	678688	ICWHOIW
hscCp7	-	reverse	682817	ICWHOIW
ybeZp	-	reverse	692692	AIPPIW, IDAIS, TIMIS
glnSp2	-	forward	705222	IDAIS
ybfEp	-	reverse	711162	AIPPIW
rhsCp3	-	forward	728602	ICWHOIW
mngAp	-	forward	765034	HIPPIW, ICIW
ybgEp4	-	forward	773373	ICWHOIW
ybgCp6	-	forward	773867	ICWHOIW
tolBp3	-	forward	776783	ICWHOIW
pglp2	-	forward	797762	ICWHOIW
ybiAp6	-	reverse	832183	ICWHOIW
ybiPp5	-	reverse	851849	ICWHOIW
fsaAp6	-	forward	862753	ICWHOIW
ybjEp5	-	reverse	914300	ICWHOIW
macBp1	-	forward	919317	AIPPIW
macBp2	-	forward	919560	AIPPIW
ycalp6	-	forward	963328	ICWHOIW

yccSp3	-	reverse	1023199	ICWHOIW
insFp5	-	reverse	1094378	ICWHOIW
ycdUp11	-	forward	1094982	ICWHOIW
yceJp	-	reverse	1118294	AIPPIW, IDAIS
yceFp8	-	reverse	1145830	ICWHOIW
pabCp8	-	forward	1152316	ICWHOIW
ycfZp6	-	reverse	1180569	ICWHOIW
ycfZp5	-	reverse	1180603	ICWHOIW
phoPp3	-	reverse	1189714	AIPPIW, IDAIS, TIMIS
croEp4	-	forward	1202106	ICWHOIW
cohEp10	-	reverse	1202311	ICWHOIW
yciAp8	-	reverse	1310438	ICWHOIW
yciEp7	-	reverse	1313345	ICWHOIW
trpAp7	-	reverse	1315363	ICWHOIW
yciNp8	-	reverse	1328726	ICWHOIW
topAp1	-	forward	1329004	HIPPIW, IMPIW, TIMIS
lapAp1	-	forward	1338159	AIPPIW, IDAIS, TIMIS
ymjAp5	-	reverse	1355834	ICWHOIW
ycjXp	-	forward	1382093	AIPPIW, IDAIS, TIMIS
mpaAp4	-	reverse	1388663	ICWHOIW
ydaSp3	-	forward	1418194	ICWHOIW
insHp3	-	reverse	1426950	ICWHOIW
ldhAp	-	reverse	1440939	AIPPIW, IDAIS, TIMIS
ynbAp3	-	forward	1475486	ICWHOIW
azoRp8	-	reverse	1481015	ICWHOIW
opgDp8	-	forward	1494679	ICWHOIW
ydcXp9	-	forward	1515378	ICWHOIW
mcbRp3	-	forward	1518120	ICWHOIW
yddEp5	-	reverse	1533893	ICWHOIW
maeAp4	-	reverse	1553751	ICWHOIW
dosPp3	-	reverse	1563767	ICWHOIW
yneJp6	-	forward	1612812	ICWHOIW
dcpp3	-	reverse	1625446	ICWHOIW
tfaQp6	-	reverse	1632980	ICWHOIW
ynfCp9	-	reverse	1655571	ICWHOIW
ydgDp4	-	forward	1669977	ICWHOIW
ydgDp7	-	forward	1669981	ICWHOIW
pntBp6	-	reverse	1674572	ICWHOIW
ydgHp5	-	forward	1676401	ICWHOIW
ydhQp	-	reverse	1744226	AIPPIW, IDAIS, TIMIS
ynhGp7	-	reverse	1756968	ICWHOIW

ynhGp4	-	reverse	1756969	ICWHOIW
ydiHp2	-	reverse	1763315	ICWHOIW
ydjOp4	-	reverse	1811372	ICWHOIW
ydjXp5	-	forward	1831241	ICWHOIW
msrBp5	-	reverse	1860608	ICWHOIW
gapAp2	-	forward	1860642	HIPPIW, IDAIS, TIMIS
yeaQp3	-	reverse	1877384	ICWHOIW
yoaBp2	-	forward	1891345	ICWHOIW
sdaAp	-	forward	1894833	HIPPIW, IDAIS, TIMIS
htpXp	-	reverse	1910642	HIPPIW, IDAIS, TIMIS
pphAp	-	reverse	1921409	AIPPIW, TIMIS
purTp5	-	forward	1928743	ICWHOIW
ruvBp1	-	reverse	1943390	ICWHOIW
ruvBp4	-	reverse	1943568	ICWHOIW
cheWp5	-	reverse	1971486	ICWHOIW
araHp7	-	reverse	1981584	ICWHOIW
yecCp3	-	reverse	1995847	ICWHOIW
yedEp8	-	forward	2006179	ICWHOIW
yodCp7	-	reverse	2026434	ICWHOIW
yodBp15	-	forward	2040200	ICWHOIW
yodBp13	-	forward	2040332	ICWHOIW
yeeJp8	-	forward	2042787	ICWHOIW
rfcp7	-	reverse	2105418	ICWHOIW
yegSp5	-	forward	2166582	ICWHOIW
mlrAp9	-	forward	2212807	ICWHOIW
sanAp3	-	forward	2230706	ICWHOIW
mglCp5	-	reverse	2235880	ICWHOIW
yeiEp5	-	reverse	2247733	ICWHOIW
yeiQp5	-	forward	2264186	ICWHOIW
rsuAp3	-	reverse	2278570	ICWHOIW
narPp	-	forward	2288444	AIPPIW, IDAIS, TIMIS
yfaEp2	-	forward	2346360	ICWHOIW
yfbLp3	-	forward	2383782	ICWHOIW
alaAp9	-	forward	2405430	ICWHOIW
yfbRp3	-	forward	2406853	ICWHOIW
dedDp2	-	reverse	2429890	ICWHOIW
dedAp2	-	reverse	2432962	ICWHOIW
smrBp4	-	forward	2446590	ICWHOIW
yfdFp4	-	forward	2460824	ICWHOIW
yfdPp5	-	forward	2471444	ICWHOIW
yfeHp3	-	forward	2524955	ICWHOIW

yffOp8	-	forward	2559924	ICWHOIW
pbpCp2	-	reverse	2645390	ICWHOIW
ryfAp11	-	forward	2651721	ICWHOIW
yfhRp4	-	forward	2662226	ICWHOIW
hcaTp6	-	reverse	2665950	ICWHOIW
hcaTp7	-	reverse	2666075	ICWHOIW
yphBp3	-	reverse	2672810	ICWHOIW
pgpCp	-	reverse	2696660	AIPPIW
rrsGp1	-	reverse	2729470	HIPPIW
clpBp	-	reverse	2732227	HIPPIW, IDAIS, TIMIS
raiAp	-	forward	2735126	IDAIS, IEPIW
yfiRp7	-	forward	2739699	ICWHOIW
ypjDp4	-	forward	2745868	ICWHOIW
grpEp	-	reverse	2748769	HIPPIW, IDAIS, TIMIS
yfjIp10	-	forward	2756950	ICWHOIW
rnlAp	-	forward	2763928	AIPPIW, IDAIS
yfjPp8	-	forward	2765562	ICWHOIW
csiRp5	-	forward	2793665	ICWHOIW
pncCp	-	reverse	2822416	HIPPIW, TIMIS
ygbLp5	-	forward	2861559	ICWHOIW
ygbLp4	-	forward	2861591	ICWHOIW
cysCp3	-	reverse	2872050	ICWHOIW
ygbFp	-	reverse	2876937	AIPPIW, TIMIS
sydp2	-	reverse	2923391	ICWHOIW
ygdIp7	-	reverse	2941251	ICWHOIW
ygdIp10	-	reverse	2941285	ICWHOIW
lplTp3	-	reverse	2971961	ICWHOIW
yqeGp8	-	forward	2983841	ICWHOIW
yqeCp5	-	reverse	3013256	ICWHOIW
idip10	-	forward	3030992	ICWHOIW
xerDp	-	reverse	3037873	HIPPIW, IDAIS
yqgBp4	-	reverse	3084204	ICWHOIW
ygglp5	-	forward	3087771	ICWHOIW
yqgEp3	-	forward	3090906	ICWHOIW
rdgBp	-	forward	3094642	AIPPIW, TIMIS
yghDp4	-	reverse	3109329	ICWHOIW
yghJp7	-	reverse	3117288	ICWHOIW
yghQp8	-	reverse	3130545	ICWHOIW
yghQp7	-	reverse	3130547	ICWHOIW
yghTp4	-	forward	3131999	ICWHOIW
yqhAp7	-	reverse	3147597	ICWHOIW

yqhHp4	-	forward	3156443	ICWHOIW
ftsPp2	-	reverse	3160888	ICWHOIW
ygiVp3	-	reverse	3167313	ICWHOIW
ygiWp7	-	reverse	3167710	ICWHOIW
ygiWp5	-	reverse	3167909	ICWHOIW
glnEp5	-	reverse	3197775	ICWHOIW
rpoDp3	-	forward	3210710	HIPPIW, IDAIS, TIMIS
ygjQp3	-	forward	3234421	ICWHOIW
exuRp	-	forward	3244530	AIPPIW
yqjFp3	-	forward	3248494	ICWHOIW
yqjFp4	-	forward	3248497	ICWHOIW
yhaKp3	-	forward	3252313	ICWHOIW
rsmIp8	-	reverse	3291575	ICWHOIW
yhcAp11	-	forward	3360000	ICWHOIW
yhcHp2	-	reverse	3367584	ICWHOIW
aaeBp7	-	reverse	3386258	ICWHOIW
tldDp6	-	reverse	3390097	ICWHOIW
yhdJp2	-	forward	3409639	ICWHOIW
yhdVp9	-	forward	3416218	ICWHOIW
rrsDp1	-	reverse	3427069	HIPPIW, TIMIS
rsmBp4	-	forward	3433013	ICWHOIW
tusBp7	-	reverse	3473131	ICWHOIW
slyXp4	-	forward	3475453	ICWHOIW
frlAp17	-	forward	3497781	ICWHOIW
frlAp5	-	forward	3497792	ICWHOIW
hslRp	-	forward	3527132	AIPPIW, TIMIS
greBp7	-	forward	3534802	ICWHOIW
yhgAp6	-	forward	3541169	ICWHOIW
nfuAp2	-	forward	3543480	AIPPIW, TIMIS
glgPp1	-	reverse	3564796	ICWHOIW
gntUp2	-	reverse	3575108	ICWHOIW
yhhXp4	-	reverse	3578882	ICWHOIW
yhhAp6	-	forward	3584752	ICWHOIW
dcrBp6	-	forward	3607953	ICWHOIW
yhhJp3	-	reverse	3624871	ICWHOIW
prlCp	-	reverse	3643261	AIPPIW, IDAIS, IEPIW
yhjVp3	-	forward	3698421	ICWHOIW
yiaDp4	-	forward	3714379	ICWHOIW
rfaDp3	-	forward	3791888	TIMIS
mutMp	-	reverse	3809198	AIPPIW, TIMIS
mutMp2	-	reverse	3809300	AIPPIW

pyrEp4	-	reverse	3814008	ICWHOIW
ligBp4	-	reverse	3819311	ICWHOIW
yidFp5	-	reverse	3853813	ICWHOIW
cbrAp6	-	forward	3867337	ICWHOIW
rarDp4	-	reverse	4002264	ICWHOIW
rarDp5	-	reverse	4002320	ICWHOIW
pldBp6	-	forward	4007037	ICWHOIW
rrsAp1	-	forward	4033262	HIPPIW
yihAp9	-	reverse	4049008	ICWHOIW
hslVp	-	reverse	4120377	HIPPIW, IDAIS, TIMIS
rpmEp	-	forward	4124931	HTTIMIS, IDAIS, TIMIS
gldAp4	-	reverse	4137069	ICWHOIW
pflDp2	-	forward	4141903	ICWHOIW
yijOp5	-	reverse	4146452	ICWHOIW
trmAp	-	reverse	4161318	HIPPIW, TIMIS
rrsBp1	-	forward	4164390	HIPPIW, TIMIS
yjaZp	-	forward	4187776	HIPPIW, TIMIS
rrsEp	-	forward	4205886	HIPPIW
metAp1	-	forward	4212256	TIMIS
zurp7	-	reverse	4258137	ICWHOIW
dusAp9	-	forward	4259623	ICWHOIW
yjcDp7	-	forward	4276337	ICWHOIW
yjcOp4	-	reverse	4295157	ICWHOIW
yjcOp5	-	reverse	4295245	ICWHOIW
alsKp1	-	reverse	4306018	ICWHOIW
alsEp3	-	reverse	4306690	ICWHOIW
adiCp7	-	reverse	4335225	ICWHOIW
groSp	-	forward	4368639	AIPPIW, HIPPIW, IDAIS
yjeJp7	-	reverse	4372392	ICWHOIW
mutLp2	-	forward	4395069	HIPPIW, IDAIS, TIMIS
miaAp2	-	forward	4397005	HIPPIW, IDAIS, TIMIS
yjfZp7	-	reverse	4425549	ICWHOIW
holCp	-	reverse	4482296	AIPPIW, IDAIS, TIMIS
yjhBp2	-	forward	4502043	ICWHOIW
fecEp3	-	reverse	4509553	ICWHOIW
fecEp4	-	reverse	4509667	ICWHOIW
yjiLp2	-	reverse	4562816	ICWHOIW
yjiLp3	-	reverse	4562825	ICWHOIW
yjiAp6	-	reverse	4587018	ICWHOIW
lplAp2	-	reverse	4622261	ICWHOIW
sltp7	-	forward	4628723	ICWHOIW

[B] Promoters identified as the constitutive promoters by SELEX

ybeDp	20.1	reverse	661929	HIPPIW, IDAIS, IEPIW
fxsAp	19.2	forward	4366626	HIPPIW, IDAIS, TIMIS
rlmEp2	14.1	reverse	3325753	HIPPIW, IDAIS, TIMIS
bssSp2	5.4	reverse	1120255	AIPPIW, IDAIS, TIMIS
bssSp1	5.4	reverse	1120281	AIPPIW, IDAIS, IEPIW
ibpAp	4.6	reverse	3865541	HIPPIW, IDAIS, TIMIS
ycjZp7	4.4	forward	1389896	ICWHOIW
ycjYp6	4.4	reverse	1390096	ICWHOIW
canp	3.9	reverse	142703	AIPPIW, HTTIMIS, IDAIS
rrsCp1	3.6	forward	3939539	AIPPIW, HIPPIW
creAp2	3.3	forward	4633266	HIPPIW, TIMIS
cldp7	3.2	reverse	2096437	ICWHOIW
ydfAp6	3.2	forward	1646481	ICWHOIW
dnaKp1	3.2	forward	12048	HIPPIW, IDAIS, TIMIS
dnaKp2	3.2	forward	12123	HIPPIW, IDAIS, TIMIS
dnaKp3	3.2	forward	12144	HIPPIW, IDAIS, TIMIS
ybgSp8	3.0	reverse	784719	ICWHOIW
yneKp6	3.0	forward	1613574	ICWHOIW
iapp7	2.9	forward	2874558	ICWHOIW
yhdNp	2.9	reverse	3437568	AIPPIW, IDAIS, TIMIS
rfbXp5	2.8	reverse	2107700	ICWHOIW
ackAp	2.7	forward	2411481	AIPPIW
hspQp	2.7	reverse	0	IDAIS, IEPIW
ygeWp6	2.7	forward	3004139	ICWHOIW
ygeWp5	2.7	forward	3004140	ICWHOIW
yagNp4	2.6	reverse	294895	ICWHOIW
prfCp2	2.5	forward	4607367	ICWHOIW
yhcOp3	2.5	reverse	3384325	ICWHOIW
ynfAp4	2.4	reverse	1653723	ICWHOIW
yjfPp4	2.3	forward	4414779	ICWHOIW
yibAp	2.2	forward	0	IDAIS, IEPIW
mppAp4	2.2	forward	1391228	ICWHOIW
yedYp4	2.2	forward	2037310	ICWHOIW
yihVp6	2.2	forward	4071555	ICWHOIW
ypjMp	2.2	reverse	0	IDAIS, IEPIW
yfjXp2	2.2	forward	2773910	ICWHOIW
yjgBp9	2.2	reverse	4494370	ICWHOIW
yjgBp8	2.2	reverse	4494372	ICWHOIW
cspHp8	2.1	reverse	1050428	ICWHOIW

cspHp12	**2.1**	**reverse**	**1050507**	**ICWHOIW**
ycaKp4	**2.0**	**forward**	**948797**	**ICWHOIW**
