# Supplementary material for: The whole set of the constitutive promoters recognized by four minor sigma subunits of Escherichia coli RNA polymerase
Source: PLoS One. 2017 Jun 30;12(6):e0179181. doi: 10.1371/journal.pone.0179181 (PMC5493296; doi:10.1371/journal.pone.0179181)
Supplement: S3 Table — Promoters listed in RegulonDB are classified into those not identified as the constitutive promoters (A) and the constitutive promoters identified by SELEX screening (B). Evidence for each promoter are as described in S1 Table. (PDF) [file pone.0179181.s003.pdf]

S3 Table
RpoF promoters (RegulonDB)

[A] Promoters not identified as the constitutive promoters

Promoter	SELEX	Direction	Genome position	Evidence
yjcSp	-	reverse	0	HIPPIW
fliDp1	-	forward	0	HIPPIW
insLp6	-	forward	15436	ICWHOIW
caiEp7	-	reverse	35399	ICWHOIW
caiEp3	-	reverse	35499	ICWHOIW
hemLp1	-	reverse	174902	HIPPIW, IEPIW
hemLp	-	reverse	174921	TIMIS
hemLp2	-	reverse	174941	HIPPIW
dxrp4	-	forward	193338	ICWHOIW
ykfBp1	-	reverse	265382	HIPPIW
ykfBp2	-	reverse	265451	HIPPIW
yagGp9	-	forward	284431	ICWHOIW
ykgCp8	-	reverse	319312	ICWHOIW
yaiIp3	-	forward	404947	ICWHOIW
ampGp6	-	reverse	452908	ICWHOIW
ybaYp3	-	forward	474469	ICWHOIW
ybaMp3	-	reverse	489548	ICWHOIW
ybaNp9	-	forward	490103	ICWHOIW
ybbJp1	-	reverse	514301	ICWHOIW
ybdJp5	-	reverse	605487	ICWHOIW
insLp5	-	forward	607279	ICWHOIW
rnkp7	-	reverse	643403	ICWHOIW
cobCp6	-	reverse	669269	ICWHOIW
ybfAp8	-	forward	728305	ICWHOIW
galMp4	-	reverse	788121	ICWHOIW
ybhNp5	-	reverse	821871	ICWHOIW
ybiXp2	-	reverse	838497	ICWHOIW
ybiWp4	-	reverse	862006	ICWHOIW
clpSp7	-	forward	922135	ICWHOIW
ycaIp8	-	forward	963331	ICWHOIW
torTp7	-	forward	1055430	ICWHOIW
ymdAp9	-	forward	1104635	ICWHOIW
yceIp5	-	reverse	1117864	ICWHOIW
lolCp6	-	forward	1174597	ICWHOIW
ycfDp5	-	reverse	1187493	ICWHOIW

nudJp6	-	reverse	1193660	ICWHOIW
ymgEp7	-	forward	1243778	ICWHOIW
lolBp	-	reverse	1262779	HIPPIW
trpAp8	-	reverse	1315265	ICWHOIW
pgpBp8	-	forward	1337206	ICWHOIW
insHp7	-	forward	1393995	ICWHOIW
ydcFp4	-	forward	1485255	ICWHOIW
trgp1	-	forward	1490463	HIPPIW, HTTIMIS
yncDp11	-	reverse	1521300	ICWHOIW
vesp	-	reverse	1823008	TIMIS
ynjFp6	-	reverse	1839635	ICWHOIW
ynjHp	-	reverse	1840187	AIPPIW, HIPPIW
ynjHp2	-	reverse	1840212	HIPPIW
dmlAp4	-	forward	1879736	ICWHOIW
yoaCp7	-	reverse	1892136	ICWHOIW
prcp1	-	reverse	1913051	ICWHOIW
tarp	-	reverse	1970740	HIPPIW, TIMIS
flhCp3	-	reverse	1975956	ICWHOIW
yecJp4	-	reverse	1985826	ICWHOIW
yecFp	-	forward	1993741	HIPPIW
fliCp	-	reverse	2001700	FPIS, HIPPIW
fliFp	-	forward	2011229	HIPPIW
fliLp2	-	forward	2017619	HIPPIW, TIMIS
yedVp6	-	reverse	2036226	ICWHOIW
yodBp14	-	forward	2040285	ICWHOIW
insHp4	-	reverse	2065535	ICWHOIW
yeiRp3	-	forward	2265764	ICWHOIW
ecop7	-	forward	2301873	ICWHOIW
rcsCp5	-	reverse	2318001	ICWHOIW
yfaDp5	-	forward	2354712	ICWHOIW
yfdPp9	-	forward	2471594	ICWHOIW
insLp5	-	forward	2512344	ICWHOIW
yfeXp5	-	reverse	2548700	ICWHOIW
yfeZp3	-	reverse	2549943	ICWHOIW
ppkp5	-	forward	2620896	ICWHOIW
pbpCp1	-	reverse	2645515	ICWHOIW
yphBp2	-	reverse	2672817	ICWHOIW
kgtPp7	-	reverse	2723782	ICWHOIW
yfiRp8	-	forward	2739724	ICWHOIW
ygbKp	-	forward	2860219	HIPPIW
ispFp2	-	reverse	2869933	ICWHOIW

ygdGp2	-	forward	2928895	ICWHOIW
ygdRp5	-	forward	2969269	ICWHOIW
yqeIp8	-	forward	2986437	ICWHOIW
yggLp3	-	reverse	3100276	ICWHOIW
glcAp12	-	reverse	3119343	ICWHOIW
aerp	-	reverse	3217142	HIPPIW, TIMIS
ygjQp6	-	forward	3234484	ICWHOIW
alxp3	-	forward	3236577	ICWHOIW
rsmIp6	-	reverse	3291415	ICWHOIW
yraQp4	-	reverse	3296229	ICWHOIW
nlpIp2	-	reverse	3307062	ICWHOIW
yhcFp5	-	forward	3364911	ICWHOIW
yhcMp4	-	reverse	3378141	ICWHOIW
tldDp7	-	reverse	3390206	ICWHOIW
rrfFp2	-	reverse	3421729	ICWHOIW
slyDp5	-	reverse	3476562	ICWHOIW
nudEp9	-	reverse	3524351	ICWHOIW
yrfFp12	-	forward	3524425	ICWHOIW
hslOp3	-	forward	3527755	ICWHOIW
rtcAp6	-	reverse	3554970	ICWHOIW
rsmDp11	-	forward	3602338	ICWHOIW
dcrBp8	-	forward	3607936	ICWHOIW
yhiMp10	-	forward	3632658	ICWHOIW
yhjHp	-	reverse	3677252	AIPPIW, HIPPIW
ghrBp2	-	forward	3715212	ICWHOIW
glmSp2	-	reverse	3911834	ICWHOIW
hsrAp6	-	reverse	3938729	ICWHOIW
aspTp1	-	forward	3944803	ICWHOIW
trpTp2	-	forward	3944803	ICWHOIW
hdfRp6	-	reverse	3946183	ICWHOIW
yifBp9	-	forward	3948007	ICWHOIW
rhtCp2	-	forward	4005762	ICWHOIW
ubiEp4	-	forward	4016770	ICWHOIW
yihOp3	-	reverse	4063994	ICWHOIW
frvRp4	-	reverse	4087925	ICWHOIW
pflDp4	-	forward	4141881	ICWHOIW
yjaHp1	-	forward	4198534	ICWHOIW
purDp1	-	reverse	4204009	ICWHOIW
yjaAp4	-	forward	4211143	ICWHOIW
rluFp6	-	forward	4228185	ICWHOIW
yjdAp2	-	forward	4325087	HIPPIW

yjdAp1	-	forward	4325102	HIPPIW
ytflp11	-	forward	4435640	ICWHOIW
yjgAp11	-	reverse	4456069	ICWHOIW
yjgAp9	-	reverse	4456088	ICWHOIW
yjgAp5	-	reverse	4456104	ICWHOIW
tabAp5	-	forward	4472806	ICWHOIW
yjhUp8	-	reverse	4518563	ICWHOIW

[B] Promoters identified as the constitutive promoters by SELEX

fliAp2	46.2	reverse	1999832	HIPPIW, TIMIS
flgKp	27.2	forward	1137574	HIPPIW
flxAp	23.2	forward	1644412	AIPPIW, HIPPIW
flgMp	15.0	reverse	1129388	HIPPIW
modAp1	9.2	forward	794218	HIPPIW
yecHp5	7.9	reverse	1987676	ICWHOIW
tsrp	5.3	forward	4589656	HIPPIW
yhiLp	4.8	reverse	0	HIPPIW
ycgRp	4.6	reverse	1243779	HIPPIW
greBp8	4.3	forward	3534766	ICWHOIW
chbGp5	4.2	reverse	1815336	ICWHOIW
cldp10	3.5	reverse	2096499	ICWHOIW
fliEp	3.4	reverse	2011117	HIPPIW
adhPp5	3.3	reverse	1552018	ICWHOIW
ppdAp	3.2	reverse	2962242	HIPPIW
motAp	3.1	reverse	1975326	HIPPIW
fryAp6	2.9	reverse	2502515	ICWHOIW
yjcOp3	2.9	reverse	4295353	ICWHOIW
oppAp	2.2	forward	1299109	AIPPIW, NTASIW
yjdLp4	2.1	reverse	4354530	ICWHOIW
