# Supplementary material for: The whole set of the constitutive promoters recognized by four minor sigma subunits of Escherichia coli RNA polymerase
Source: PLoS One. 2017 Jun 30;12(6):e0179181. doi: 10.1371/journal.pone.0179181 (PMC5493296; doi:10.1371/journal.pone.0179181)
Supplement: S4 Table — Promoters listed in RegulonDB are classified into those not identified as the constitutive promoters (A) and the constitutive promoters identified by SELEX screening (B). Evidence for each promoter are as described in S1 Table. (PDF) [file pone.0179181.s004.pdf]

S4 Table
RpoE promoters (RegulonDB)

[A] Promoters not identified as the constitutive promoters

Promoter	SELEX	Direction	Genome position	Evidence
surAp	-	reverse	0	NTASIW, TASESIW
yaaAp3	-	reverse	6587	ICWHOIW
apaHp4	-	reverse	51293	ICWHOIW
impp3	-	reverse	57336	HIPPIW, TIMIS
rluAp1	-	reverse	60450	ICWHOIW
leuDp4	-	reverse	79594	ICWHOIW
ampDp5	-	forward	118681	ICWHOIW
yadEp3	-	forward	144965	ICWHOIW
panCp2	-	reverse	148920	ICWHOIW
yadNp1	-	reverse	156984	ICWHOIW
gluQp1	-	reverse	160143	ICWHOIW
yadSp7	-	reverse	177757	ICWHOIW
degPp	-	forward	180845	HIPPIW, TIMIS
frrp6	-	forward	192849	ICWHOIW
dxrp5	-	forward	193411	ICWHOIW
dxrp1	-	forward	193471	ICWHOIW
rsePp	-	forward	196470	HIPPIW, TIMIS
bamAp2	-	forward	197026	AIPPIW
bamAp	-	forward	197821	HIPPIW, TIMIS
hlpAp	-	forward	200455	HIPPIW, TIMIS
lpxDp	-	forward	200960	HIPPIW, TIMIS
yaeFp1	-	reverse	217135	ICWHOIW
lpcAp1	-	forward	243468	ICWHOIW
ykfIp7	-	reverse	263042	ICWHOIW
mmuPp1	-	forward	274343	ICWHOIW
mmuPp2	-	forward	274539	ICWHOIW
ykgJp2	-	reverse	303482	ICWHOIW
matAp1	-	reverse	310617	ICWHOIW
matAp5	-	reverse	310670	ICWHOIW
ykgLp1	-	forward	311163	ICWHOIW
ykgBp3	-	reverse	317600	ICWHOIW
yaiOp6	-	reverse	380074	ICWHOIW
yaiPp5	-	reverse	383209	ICWHOIW
yaiSp13	-	reverse	383962	ICWHOIW
insFp4	-	reverse	391884	ICWHOIW

sbmAp	-	forward	395777	AIPPIW, HIPPIW, TIMIS
yaiIp7	-	forward	404922	ICWHOIW
sbcCp2	-	reverse	415071	ICWHOIW
yajIp6	-	reverse	432258	ICWHOIW
ampGp5	-	reverse	452851	ICWHOIW
clpXp	-	forward	456426	AIPPIW, TIMIS
fadMp1	-	forward	463584	ICWHOIW
ybaNp1	-	forward	489942	ICWHOIW
ybaBp	-	forward	492933	AIPPIW, TIMIS
ybaPp6	-	reverse	507508	ICWHOIW
ylbAp2	-	reverse	543280	ICWHOIW
fimZp3	-	reverse	563830	ICWHOIW
intDp6	-	reverse	565324	ICWHOIW
essDp	-	forward	576096	AIPPIW, TIMIS
ybcWp4	-	forward	579028	ICWHOIW
ybcHp4	-	reverse	587293	ICWHOIW
ybdFp5	-	reverse	605276	ICWHOIW
ybdKp3	-	reverse	606784	ICWHOIW
hokEp	-	forward	606954	AIPPIW
ybdDp2	-	forward	631214	ICWHOIW
ybdMp2	-	reverse	634778	ICWHOIW
ahpFp	-	forward	638225	AIPPIW, TIMIS
citTp4	-	reverse	645855	ICWHOIW
crcBp4	-	reverse	657231	ICWHOIW
lipBp2	-	reverse	661624	ICWHOIW
dacAp2	-	reverse	663190	ICWHOIW
cobCp7	-	reverse	669167	ICWHOIW
ybeRp7	-	forward	675783	ICWHOIW
ybeQp1	-	reverse	675929	ICWHOIW
rihAp3	-	reverse	683825	ICWHOIW
insHp3	-	reverse	688428	ICWHOIW
lntp4	-	reverse	690207	ICWHOIW
ybfFp1	-	reverse	712069	ICWHOIW
ybfHp	-	reverse	715864	AIPPIW, TIMIS
potEp1	-	reverse	717567	ICWHOIW
ybfAp7	-	forward	728260	ICWHOIW
ybgOp4	-	reverse	748405	ICWHOIW
ybgDp9	-	reverse	752181	ICWHOIW
ybgSp2	-	reverse	784682	ICWHOIW
ybhHp8	-	forward	799775	ICWHOIW
rlmFp1	-	forward	841415	ICWHOIW

ybiUp5	-	reverse	858323	ICWHOIW
ybjOp4	-	forward	897075	ICWHOIW
hcrp3	-	reverse	911423	ICWHOIW
hcpp2	-	reverse	913352	AIPPIW, TIMIS
ybjEp3	-	reverse	914124	ICWHOIW
aqpZp2	-	reverse	915435	ICWHOIW
cydCp5	-	reverse	928623	ICWHOIW
ycaLp1	-	forward	959355	ICWHOIW
ycaLp8	-	forward	959366	ICWHOIW
elfAp6	-	forward	996988	ICWHOIW
elfDp1	-	forward	997544	ICWHOIW
yccXp7	-	forward	1029217	ICWHOIW
gfcAp1	-	reverse	1049057	ICWHOIW
torTp1	-	forward	1055443	ICWHOIW
torTp2	-	forward	1055445	ICWHOIW
rutGp2	-	reverse	1069268	ICWHOIW
rutRp	-	forward	1073357	AIPPIW, TIMIS
insFp4	-	reverse	1094419	ICWHOIW
ycdUp9	-	forward	1094856	ICWHOIW
ycdUp1	-	forward	1094916	ICWHOIW
csgGp1	-	reverse	1100978	ICWHOIW
ymdBp4	-	forward	1104961	ICWHOIW
yceOp1	-	reverse	1118734	ICWHOIW
yceOp7	-	reverse	1118855	ICWHOIW
yceHp2	-	forward	1125304	ICWHOIW
yceMp4	-	forward	1125827	ICWHOIW
flgNp1	-	reverse	1129137	ICWHOIW
yceFp9	-	reverse	1145972	ICWHOIW
pabCp5	-	forward	1152417	ICWHOIW
pabCp1	-	forward	1152516	ICWHOIW
bhsAp	-	forward	1168256	HTTIMIS
ycfTp9	-	reverse	1174548	ICWHOIW
ycfTp11	-	reverse	1174552	ICWHOIW
phoQp5	-	reverse	1189163	ICWHOIW
litp1	-	forward	1197739	ICWHOIW
ymflp1	-	forward	1200663	ICWHOIW
ymfJp9	-	reverse	1201482	ICWHOIW
tfaEp6	-	reverse	1208412	ICWHOIW
tfaEp1	-	reverse	1208489	ICWHOIW
mcrAp7	-	forward	1209362	ICWHOIW
mcrAp10	-	forward	1209418	ICWHOIW

minEp3	-	reverse	1223934	ICWHOIW
ldcAp9	-	reverse	1242420	ICWHOIW
ymgEp6	-	forward	1243802	ICWHOIW
ycgYp1	-	forward	1244306	ICWHOIW
ycgYp6	-	forward	1244324	ICWHOIW
ychNp4	-	reverse	1272878	ICWHOIW
tdkp1	-	forward	1292739	ICWHOIW
yciUp5	-	reverse	1305376	ICWHOIW
yciBp3	-	reverse	1311079	ICWHOIW
yciEp6	-	reverse	1313262	ICWHOIW
yciNp9	-	reverse	1328781	ICWHOIW
pgpBp5	-	forward	1337217	ICWHOIW
yciTp1	-	reverse	1342568	ICWHOIW
yciWp4	-	reverse	1348260	ICWHOIW
ycjDp4	-	reverse	1349890	ICWHOIW
sapFp5	-	reverse	1350681	ICWHOIW
ycjMp4	-	forward	1368027	ICWHOIW
smrAp1	-	forward	1403960	ICWHOIW
dbpAp2	-	forward	1407362	ICWHOIW
intRp4	-	reverse	1411284	ICWHOIW
racCp4	-	reverse	1415835	ICWHOIW
racCp6	-	reverse	1415839	ICWHOIW
pinRp1	-	reverse	1431736	ICWHOIW
ydbJp1	-	forward	1438880	ICWHOIW
azoRp1	-	reverse	1480897	ICWHOIW
azoRp6	-	reverse	1480951	ICWHOIW
ydcAp6	-	forward	1489671	ICWHOIW
ydcAp7	-	forward	1489677	ICWHOIW
opgDp4	-	forward	1494669	ICWHOIW
ydcKp8	-	reverse	1498664	ICWHOIW
ydcLp7	-	forward	1500347	ICWHOIW
patDp3	-	forward	1513439	ICWHOIW
patDp4	-	forward	1513464	ICWHOIW
ydcXp1	-	forward	1515245	ICWHOIW
ydcZp1	-	reverse	1516399	ICWHOIW
yncBp4	-	forward	1516916	ICWHOIW
pptAp7	-	forward	1530882	ICWHOIW
nhoAp2	-	forward	1531987	ICWHOIW
narWp	-	reverse	1536711	AIPPIW, TIMIS
gadCp5	-	reverse	1568551	ICWHOIW
gadCp6	-	reverse	1568601	ICWHOIW

gadCp1	-	reverse	1568608	ICWHOIW
yddBp6	-	reverse	1575690	ICWHOIW
yneEp5	-	reverse	1607206	ICWHOIW
yneFp7	-	reverse	1609898	ICWHOIW
sadp5	-	reverse	1612823	ICWHOIW
ydeIp1	-	reverse	1622540	ICWHOIW
ydfKp5	-	forward	1631009	ICWHOIW
pinQp1	-	forward	1631608	ICWHOIW
ynfOp1	-	forward	1634751	ICWHOIW
remp1	-	reverse	1642996	ICWHOIW
ynfAp7	-	reverse	1653703	ICWHOIW
ynfDp6	-	forward	1655438	ICWHOIW
ydhIp	-	forward	1719022	AIPPIW, TIMIS
ydhFp1	-	reverse	1723672	ICWHOIW
lhrp	-	forward	1726377	AIPPIW, TIMIS
mdtKp10	-	forward	1741293	ICWHOIW
ydhSp3	-	forward	1745092	ICWHOIW
ydhSp6	-	forward	1745106	ICWHOIW
ydhSp5	-	forward	1745137	ICWHOIW
ydhTp1	-	reverse	1747593	ICWHOIW
ydiMp5	-	forward	1768950	ICWHOIW
ydiMp1	-	forward	1768954	ICWHOIW
ppsRp1	-	forward	1785363	ICWHOIW
ydiVp	-	reverse	1790111	AIPPIW
nlpCp3	-	reverse	1790851	ICWHOIW
ydiYp6	-	reverse	1804241	ICWHOIW
chbGp4	-	reverse	1815266	ICWHOIW
astEp3	-	reverse	1825093	ICWHOIW
ydjXp1	-	forward	1831332	ICWHOIW
nudGp3	-	forward	1839462	ICWHOIW
ynjIp1	-	reverse	1843035	ICWHOIW
ydjFp1	-	reverse	1852948	ICWHOIW
yeaIp6	-	forward	1868267	ICWHOIW
yeaNp4	-	forward	1873487	ICWHOIW
yeaMp1	-	reverse	1873672	ICWHOIW
yeaOp4	-	forward	1874801	ICWHOIW
yeaQp1	-	reverse	1877414	ICWHOIW
yeaYp	-	reverse	1888585	AIPPIW, HIPPIW, TIMIS
yoaCp1	-	forward	1891990	ICWHOIW
yoaHp1	-	reverse	1892957	ICWHOIW
kdgRp8	-	reverse	1908149	ICWHOIW

kdgRp1	-	reverse	1908277	ICWHOIW
yebVp1	-	forward	1919769	ICWHOIW
yebVp5	-	forward	1919777	ICWHOIW
yebWp3	-	forward	1919981	ICWHOIW
yebAp1	-	reverse	1939707	ICWHOIW
yebAp6	-	reverse	1939745	ICWHOIW
yebAp5	-	reverse	1939857	ICWHOIW
yecDp4	-	forward	1948802	ICWHOIW
micLp	-	reverse	1956771	TIMIS
yedJp3	-	reverse	2031227	ICWHOIW
yodBp16	-	forward	2040318	ICWHOIW
mtfAp1	-	forward	2041642	ICWHOIW
yeeOp1	-	reverse	2057852	ICWHOIW
yeeOp6	-	reverse	2057899	ICWHOIW
yeeAp6	-	reverse	2078685	ICWHOIW
yeeFp2	-	reverse	2085262	ICWHOIW
yeeZp7	-	reverse	2087200	ICWHOIW
wbbKp4	-	reverse	2102630	ICWHOIW
wcaMp1	-	reverse	2113958	ICWHOIW
wzap2	-	reverse	2135217	AIPPIW, TIMIS
cyaRp	-	forward	2165138	AIPPIW, HTTIMIS, TIMIS
yegXp1	-	reverse	2181823	ICWHOIW
yegXp6	-	reverse	2181842	ICWHOIW
thiDp3	-	reverse	2182695	ICWHOIW
rcnBp	-	forward	2184906	ICWHOIW
yehEp8	-	reverse	2190923	ICWHOIW
yehEp1	-	reverse	2190931	ICWHOIW
mrpp2	-	reverse	2192197	ICWHOIW
mrpp7	-	reverse	2192202	ICWHOIW
yohCp4	-	reverse	2223691	ICWHOIW
yohCp3	-	reverse	2223701	ICWHOIW
yohDp2	-	forward	2223785	ICWHOIW
mglCp9	-	reverse	2235945	ICWHOIW
mglCp6	-	reverse	2235981	ICWHOIW
psuTp2	-	reverse	2255518	ICWHOIW
psuGp4	-	reverse	2256579	ICWHOIW
yeiWp7	-	reverse	2263435	ICWHOIW
yejKp5	-	reverse	2282131	ICWHOIW
ccmHp1	-	reverse	2290643	ICWHOIW
mqop1	-	reverse	2304946	ICWHOIW
apbEp1	-	reverse	2309616	ICWHOIW

rcsCp4	-	reverse	2318103	ICWHOIW
yfaPp4	-	reverse	2326221	ICWHOIW
yfaLp1	-	reverse	2342240	ICWHOIW
yfaLp3	-	reverse	2342287	ICWHOIW
elaAp4	-	reverse	2379708	ICWHOIW
yfbLp7	-	forward	2383850	ICWHOIW
yfbKp7	-	reverse	2383927	ICWHOIW
yfbNp1	-	reverse	2386609	ICWHOIW
yfcIp8	-	reverse	2421636	ICWHOIW
yfcIp7	-	reverse	2421734	ICWHOIW
yfcVp8	-	reverse	2453710	ICWHOIW
yfdGp4	-	forward	2465759	ICWHOIW
emrYp1	-	reverse	2480356	ICWHOIW
ypdIp1	-	forward	2492516	ICWHOIW
alaXp2	-	reverse	2516301	ICWHOIW
yfeCp4	-	forward	2516397	ICWHOIW
yfeHp5	-	forward	2524931	ICWHOIW
yfeRp1	-	reverse	2525061	ICWHOIW
cysZp1	-	forward	2529405	ICWHOIW
yfeYp	-	reverse	2549265	AIPPIW, TIMIS
intZp3	-	forward	2556715	ICWHOIW
yffOp1	-	forward	2560102	ICWHOIW
yffQp1	-	forward	2561411	ICWHOIW
yffQp3	-	forward	2561431	ICWHOIW
ypfMp2	-	reverse	2589092	ICWHOIW
bamCp	-	reverse	2597036	HIPPIW, TIMIS
bepAp1	-	forward	2614090	AIPPIW, TIMIS
ispGp1	-	reverse	2639902	ICWHOIW
ispGp4	-	reverse	2639972	ICWHOIW
ispGp5	-	reverse	2639976	ICWHOIW
iscAp7	-	reverse	2657975	ICWHOIW
iscAp6	-	reverse	2657984	ICWHOIW
yfhRp2	-	forward	2662266	ICWHOIW
hcaTp1	-	reverse	2665885	ICWHOIW
acpSp2	-	reverse	2699159	ICWHOIW
acpSp3	-	reverse	2699184	ICWHOIW
lepBp1	-	reverse	2703486	ICWHOIW
rseAp3	-	reverse	2707654	AIPPIW, TIMIS
bamDp2	-	forward	2733983	AIPPIW, TIMIS
bamDp	-	forward	2734028	HIPPIW, TIMIS
rplSp1	-	reverse	2742664	ICWHOIW

bamEp	-	forward	2751508	AIPPIW, TIMIS
yfjIp8	-	forward	2756903	ICWHOIW
rnlBp	-	forward	2764729	AIPPIW, TIMIS
csiRp3	-	forward	2793493	ICWHOIW
alaEp1	-	forward	2797092	ICWHOIW
ygbNp2	-	forward	2862929	ICWHOIW
ygcNp3	-	forward	2890594	ICWHOIW
ygcUp5	-	reverse	2897629	ICWHOIW
ygcGp7	-	forward	2903627	ICWHOIW
mazGp4	-	reverse	2908882	ICWHOIW
gudDp1	-	reverse	2917535	ICWHOIW
thyAp2	-	reverse	2963370	ICWHOIW
mutHp5	-	forward	2967604	ICWHOIW
ygeAp5	-	reverse	2978825	ICWHOIW
yqeFp1	-	reverse	2983646	ICWHOIW
yqeFp8	-	reverse	2983723	ICWHOIW
yqeGp1	-	forward	2983741	ICWHOIW
yqeFp11	-	reverse	2983831	ICWHOIW
yqeIp1	-	forward	2986366	ICWHOIW
yqeKp12	-	reverse	2988592	ICWHOIW
hyuAp6	-	forward	3007852	ICWHOIW
hyuAp1	-	forward	3007941	ICWHOIW
ygfSp5	-	reverse	3027186	ICWHOIW
ygfUp6	-	forward	3029211	ICWHOIW
yqfAp10	-	reverse	3041386	ICWHOIW
yggCp1	-	reverse	3072778	ICWHOIW
yqgBp1	-	reverse	3084272	ICWHOIW
yggIp6	-	forward	3087610	ICWHOIW
rsmEp1	-	forward	3089040	ICWHOIW
yggNp2	-	reverse	3099766	HIPPIW, TIMIS
yggNp1	-	reverse	3099820	HIPPIW, TIMIS
yqgAp4	-	forward	3107543	ICWHOIW
yqgAp1	-	forward	3107556	ICWHOIW
yghDp3	-	reverse	3109298	ICWHOIW
pppAp3	-	reverse	3112501	ICWHOIW
yghQp1	-	reverse	3130615	ICWHOIW
yghTp14	-	forward	3131937	ICWHOIW
gprp1	-	forward	3145803	ICWHOIW
yqhCp1	-	reverse	3153406	ICWHOIW
ygiQp1	-	reverse	3159332	ICWHOIW
ftsPp1	-	reverse	3160873	ICWHOIW

mdaBp1	-	forward	3170445	ICWHOIW
ygiDp3	-	forward	3180451	ICWHOIW
ygiDp2	-	reverse	3180583	ICWHOIW
yqiCp1	-	forward	3182853	ICWHOIW
glnEp7	-	reverse	3197716	ICWHOIW
glnEp1	-	reverse	3197720	ICWHOIW
ygiMp2	-	forward	3199064	AIPPIW, TIMIS
ygiMp	-	forward	3199126	HIPPIW, TIMIS
bacAp	-	reverse	3202197	AIPPIW, TIMIS
plsYp5	-	forward	3202555	ICWHOIW
rpoDp4	-	forward	3210715	AIPPIW, TIMIS
ygjIp2	-	forward	3224092	ICWHOIW
ygjIp1	-	forward	3224164	ICWHOIW
higAp8	-	reverse	3232363	ICWHOIW
rlmGp4	-	reverse	3233914	ICWHOIW
ygjQp4	-	forward	3234387	ICWHOIW
yqjAp2	-	forward	3245665	HIPPIW, TIMIS
yhaHp7	-	forward	3250271	ICWHOIW
yhaIp9	-	forward	3250722	ICWHOIW
yhaIp12	-	forward	3250847	ICWHOIW
yhaKp5	-	forward	3252291	ICWHOIW
yhaKp4	-	forward	3252296	ICWHOIW
tdcRp8	-	forward	3265276	ICWHOIW
yraHp4	-	forward	3285387	ICWHOIW
yraPp2	-	forward	3294094	AIPPIW, TIMIS
yraPp	-	forward	3294371	HIPPIW, TIMIS
yraRp1	-	reverse	3297034	ICWHOIW
yhbUp7	-	forward	3299301	ICWHOIW
yhbUp1	-	forward	3299351	ICWHOIW
deaDp1	-	reverse	3305931	ICWHOIW
ftsHp1	-	reverse	3325124	ICWHOIW
greAp2	-	reverse	3326875	AIPPIW, TIMIS
ibaGp2	-	reverse	3334908	HIPPIW, ICWHOIW
lptAp1	-	forward	3341348	HIPPIW, IDAIS, TIMIS
lptBp	-	forward	3341418	AIPPIW, TIMIS
yhcAp1	-	forward	3360061	ICWHOIW
yhcFp1	-	forward	3364742	ICWHOIW
yhcMp1	-	reverse	3378178	ICWHOIW
aaeBp8	-	reverse	3386280	ICWHOIW
aaeBp3	-	reverse	3386285	ICWHOIW
yhdHp1	-	forward	3401302	ICWHOIW

yhdUp1	-	forward	3410519	ICWHOIW
envRp11	-	reverse	3411509	ICWHOIW
envRp13	-	reverse	3411623	ICWHOIW
gspAp4	-	reverse	3453656	AIPPIW, TIMIS
fusAp	-	reverse	3471707	AIPPIW, TIMIS
fkpAp1	-	reverse	3475547	AIPPIW, IHBCEIW, TIMIS
yhfYp1	-	reverse	3509639	ICWHOIW
yhfYp3	-	reverse	3509644	ICWHOIW
nudEp1	-	reverse	3524208	ICWHOIW
yrfFp6	-	forward	3524296	ICWHOIW
yhgFp2	-	forward	3535198	ICWHOIW
malQp	-	reverse	3548421	AIPPIW, TIMIS
rtcAp1	-	reverse	3554902	ICWHOIW
rtcAp2	-	reverse	3555024	ICWHOIW
glgXp3	-	reverse	3569442	ICWHOIW
ugpQp1	-	reverse	3586323	ICWHOIW
panZp1	-	forward	3595851	ICWHOIW
ftsXp3	-	reverse	3600275	ICWHOIW
ftsXp4	-	reverse	3600279	ICWHOIW
rsmDp1	-	forward	3602375	ICWHOIW
dcrBp1	-	forward	3607790	ICWHOIW
dcrBp2	-	forward	3607803	ICWHOIW
rhsBp9	-	forward	3617067	ICWHOIW
rhsBp3	-	forward	3617097	ICWHOIW
rhsBp12	-	forward	3617120	ICWHOIW
yhiMp11	-	forward	3632840	ICWHOIW
yhjRp12	-	reverse	3694238	ICWHOIW
yhjRp10	-	reverse	3694306	ICWHOIW
yhjRp5	-	reverse	3694379	ICWHOIW
yhjRp13	-	reverse	3694398	ICWHOIW
yhjXp6	-	reverse	3710120	ICWHOIW
bisCp4	-	reverse	3714438	ICWHOIW
insJp4	-	forward	3718617	ICWHOIW
hokAp6	-	reverse	3718833	ICWHOIW
insKp	-	forward	3719088	HIPPIW
ysaBp1	-	reverse	3723925	ICWHOIW
yiaBp1	-	reverse	3725820	ICWHOIW
yiaKp2	-	forward	3740743	AIPPIW, TIMIS
gpsAp8	-	reverse	3781704	ICWHOIW
yibBp6	-	reverse	3791857	ICWHOIW
waaUp9	-	reverse	3797421	ICWHOIW

yicJp	-	reverse	3834687	AIPPIW, TIMIS
yicNp4	-	reverse	3840532	ICWHOIW
yicOp11	-	reverse	3841842	ICWHOIW
yicOp5	-	reverse	3841903	ICWHOIW
yicOp1	-	reverse	3841980	ICWHOIW
yidQp	-	forward	3865567	HIPPIW, TIMIS
yieHp4	-	forward	3894680	ICWHOIW
yieKp2	-	reverse	3897487	ICWHOIW
recQp1	-	forward	4003881	ICWHOIW
yihFp4	-	forward	4042078	ICWHOIW
yihFp1	-	forward	4042198	ICWHOIW
yihLp2	-	forward	4058276	ICWHOIW
yihNp5	-	forward	4060189	ICWHOIW
yihQp1	-	reverse	4067469	ICWHOIW
cpxAp1	-	reverse	4103124	ICWHOIW
yiiSp	-	forward	4110892	AIPPIW, TIMIS
yijEp5	-	forward	4134112	ICWHOIW
arpAp1	-	reverse	4220652	ICWHOIW
rluFp1	-	forward	4228332	ICWHOIW
xylEp8	-	reverse	4240283	ICWHOIW
xylEp1	-	reverse	4240293	ICWHOIW
plsBp	-	reverse	4254621	AIPPIW, TIMIS
gltPp8	-	forward	4292291	ICWHOIW
gltPp1	-	forward	4292401	ICWHOIW
mdtPp6	-	reverse	4299259	ICWHOIW
frdDp2	-	reverse	4377456	ICWHOIW
frdDp1	-	reverse	4377496	ICWHOIW
psdp	-	reverse	4388661	AIPPIW, TIMIS
yjfPp5	-	forward	4414958	ICWHOIW
ytfHp6	-	forward	4432127	ICWHOIW
ytfJp	-	reverse	4437309	HIPPIW, TIMIS
tamAp1	-	forward	4440293	ICWHOIW
ytfQp6	-	forward	4447840	ICWHOIW
yjgZp1	-	forward	4499081	ICWHOIW
yjhBp7	-	forward	4501905	ICWHOIW
nanSp1	-	reverse	4535726	ICWHOIW
yjiJp4	-	reverse	4560705	ICWHOIW
yjiKp7	-	reverse	4561753	ICWHOIW
yjiKp9	-	reverse	4561757	ICWHOIW
hsdSp1	-	reverse	4579525	ICWHOIW
yjiAp7	-	reverse	4587071	ICWHOIW

yjiYp3	-	reverse	4589370	ICWHOIW
yjjLp7	-	reverse	4592860	ICWHOIW
yjjLp6	-	reverse	4592867	ICWHOIW
yjjLp8	-	reverse	4592905	ICWHOIW
yjjAp1	-	reverse	4598360	ICWHOIW

[B] Promoters identified as the constitutive promoters by SELEX

rybBp	55.5	reverse	887280	HIPPIW, IDAIS, TIMIS
micAp	40.9	forward	2812824	HIPPIW, TIMIS
rpoHp3	37.4	reverse	3598893	AIPPIW, HIPPIW, TIMIS
yahMp5	17.8	forward	344560	ICWHOIW
yniDp6	10.1	forward	1803125	ICWHOIW
yfeKp	7.5	forward	2535337	AIPPIW, TIMIS
cspFp8	7.3	forward	1639870	ICWHOIW
yrhBp9	6.5	forward	3582743	ICWHOIW
ymgFp1	5.4	forward	1218037	ICWHOIW
oxcp10	4.9	reverse	2490152	ICWHOIW
oxcp11	4.9	reverse	2490166	ICWHOIW
cspHp14	4.7	reverse	1050527	ICWHOIW
cspHp15	4.7	reverse	1050530	ICWHOIW
cspHp13	4.7	reverse	1050596	ICWHOIW
yjtDp8	4.6	forward	4638861	ICWHOIW
ygeWp1	4.5	forward	3004246	ICWHOIW
ydjEp5	4.4	reverse	1852007	ICWHOIW
iapp6	4.2	forward	2874566	ICWHOIW
ykgCp1	4.0	reverse	319439	ICWHOIW
ybgSp1	3.9	reverse	784562	ICWHOIW
yjjMp4	3.9	reverse	4593970	ICWHOIW
dsbCp	3.7	reverse	3036856	HIPPIW, TIMIS
dsbCp2	3.7	reverse	3037655	ICWHOIW
yniAp7	3.6	forward	1805603	ICWHOIW
yniAp1	3.6	forward	1805743	ICWHOIW
yibGp6	3.6	forward	3766087	ICWHOIW
ydhBp2	3.5	reverse	1737833	ICWHOIW
ydhCp1	3.5	forward	1737879	ICWHOIW
yghFp	3.4	reverse	3110921	HIPPIW, TIMIS
yhcOp1	3.4	reverse	3384232	ICWHOIW
setCp14	3.4	forward	3834767	ICWHOIW
yacHp3	3.3	reverse	131307	ICWHOIW
yfbPp7	3.3	forward	2387017	ICWHOIW

xanPp5	3.3	forward	3826844	ICWHOIW
lpxPp	3.3	forward	2493600	AIPPIW, HIPPIW, TIMIS
ydhZp6	3.2	reverse	1753233	ICWHOIW
yfjPp5	3.2	forward	2765664	ICWHOIW
cldp9	3.0	reverse	2096334	ICWHOIW
yjhGp4	3.0	reverse	4522249	ICWHOIW
yieEp	3.0	forward	3891852	AIPPIW, TIMIS
cybBp5	2.8	forward	1488785	ICWHOIW
cybBp7	2.8	forward	1488790	ICWHOIW
yejGp5	2.8	reverse	2276375	ICWHOIW
yejGp8	2.8	reverse	2276468	ICWHOIW
yfbOp1	2.7	forward	2386595	ICWHOIW
mppAp5	2.7	forward	1391078	ICWHOIW
eptBp	2.6	reverse	3708604	TIMIS
ygcWp11	2.6	reverse	2898431	ICWHOIW
ygcWp12	2.6	reverse	2898493	ICWHOIW
yaeQp3	2.6	forward	214088	ICWHOIW
ygeRp1	2.5	reverse	2998061	ICWHOIW
ycaDp1	2.5	forward	944942	ICWHOIW
ydeRp1	2.5	reverse	1586322	ICWHOIW
bamBp	2.5	reverse	2636997	AIPPIW, TIMIS
ulaRp5	2.5	reverse	4416585	ICWHOIW
etkp1	2.4	reverse	1043526	ICWHOIW
yfgGp7	2.3	forward	2627107	ICWHOIW
yjcEp8	2.3	forward	4277852	ICWHOIW
yjcEp7	2.3	forward	4277858	ICWHOIW
ybdHp6	2.2	reverse	632740	ICWHOIW
ypjAp8	2.2	reverse	2780844	ICWHOIW
ftnBp	2.1	forward	1984799	AIPPIW, TIMIS
yddJp8	2.1	reverse	1542782	ICWHOIW
yddJp9	2.1	reverse	1542787	ICWHOIW
yddJp1	2.1	reverse	1542890	ICWHOIW
rfaDp4	2.1	forward	3791993	HIPPIW, TIMIS
yeiIp2	2.1	forward	2249675	ICWHOIW
yhjJp	2.1	reverse	3680039	AIPPIW, TIMIS
